# Adherence to the EAT–Lancet diet and neuropsychiatric disorders: a systematic review and meta-analysis

**DOI:** 10.1017/S0033291726104528

**Published:** 2026-06-01

**Authors:** Yuhao Wang, Zejun Li, Zongze Wu, Wei Hu, Hao Wu, Yuchang Hu, Yumeng Ju, Yan Zhang

**Affiliations:** 1 Department of Psychiatry, National Clinical Research Center for Mental Disorders, and National Center for Mental Disorders, the Second Xiangya Hospital of Central South University, Changsha 410011, Hunan, China; 2 Xiangya School of Medicine, Central South University, Changsha 410011, Hunan, China, China; 3 School of Medicine, Zhejiang University, Hangzhou 310000, China; 4 School of Biological Science and Medical Engineering, Beihang University, 100191, Beijing, China

**Keywords:** EAT–Lancet diet, neuropsychiatric disorders, anxiety, depression, stroke, dementia, cognition, observational studies, planetary health diet, meta-analysis

## Abstract

Neuropsychiatric disorders (NPDs) are a leading cause of disability worldwide. The predominantly plant-based EAT–Lancet diet has been proposed to confer neuropsychiatric benefits, yet evidence remains limited. This study synthesized associations between adherence to the EAT–Lancet diet and neuropsychiatric outcomes. We searched PubMed, Web of Science, Embase, Scopus, and ProQuest Dissertations and Theses Global through September 4, 2025. Observational studies reporting associations between EAT–Lancet adherence and NPDs were included. Binary outcomes were pooled as hazard ratios (HRs) or odds ratios (ORs), and continuous outcomes as regression coefficients (β). Subgroup, sensitivity, and publication-bias analyses were performed. Certainty of evidence was assessed using the Grading of Recommendations Assessment, Development and Evaluation framework (GRADE). Twenty-two cohort and six cross-sectional studies were included. Higher adherence to the EAT–Lancet diet was associated with lower risks of depression (OR 0.76; 95% CI 0.71–0.81), anxiety (OR 0.82; 0.76–0.89), stroke (HR 0.84; 0.76–0.92), and dementia (HR 0.96; 0.93–1.00), whereas no significant association was observed for global cognitive function (β 0.02; −0.01 to 0.06). Sensitivity analyses supported robustness. Certainty of evidence was very low for anxiety, depression, and cognition, and low for stroke and dementia. Greater adherence to the EAT–Lancet diet was associated with lower risks of depression, anxiety, stroke, and dementia. However, given the low certainty of evidence, findings should be interpreted cautiously. Further large prospective studies and randomized controlled trials are warranted to improve evidence quality and clarify the potential role of the EAT–Lancet diet in neuropsychiatric disease prevention.

## Background

Neuropsychiatric disorders (NPDs), encompassing a broad spectrum of neurological and psychiatric conditions such as stroke, epilepsy, dementia, schizophrenia, depression, and anxiety, have emerged as a major global health challenge (Wang & Wang, 2025), affecting an estimated 450 million individuals worldwide (Klein, 2024). With the aging of the global population and the increasing burden of psychosocial stress, NPDs have become leading contributors to disability and mortality worldwide (Feigin et al., 2019; Whiteford et al., 2013). The World Health Organization estimates that NPDs account for at least 20% of illness-related disability globally (Reynolds et al., 2009). Furthermore, evidence has indicated that up to 90% of suicides are associated with underlying mental illness (Insel, 2009). These disorders not only impair individuals’ functional abilities and quality of life but also exert a substantial burden on families, healthcare systems, and society (Feigin et al., 2021; Ngui, Khasakhala, Ndetei, & Roberts, 2010). There is an increasingly stronger consensus that early prevention is of critical importance to reduce their incidence and long-term impact.

Among early prevention strategies, dietary patterns have gained attention as a modifiable and accessible factor in the etiology and prevention of NPDs (Molendijk et al., 2018; Sarris et al., 2015). Unhealthy dietary patterns, such as excessive consumption of refined sugars, saturated and trans fats, and ultra-processed foods, were shown to significantly increase the risks of NPDs (Huang et al., 2023; Lane et al., 2024; Sacks et al., 2017). In contrast, adherence to healthy dietary patterns that emphasize fruits, vegetables, whole grains, fish, and unsaturated fats is associated with reduced risks of NPDs (Tessier et al., 2025). Compared with many other factors that may influence the risk of neuropsychiatric disorders, including social, developmental, and genetic factors, dietary habits may be more modifiable through individual behavioral change and population-level interventions, making diet a practical and potentially scalable target for prevention (Firth et al., 2020; Townsend et al., 2023; Tsalamandris, Hadjivassiliou, & Zis, 2023).

In 2019, the EAT–Lancet Commission proposed the EAT–Lancet reference diet, also known as the Planetary Health Diet, to promote both human health and environmental sustainability (Berthy et al., 2022; Willett et al., 2019). Unlike strictly plant-based diets that exclude all animal products, the EAT–Lancet diet is a predominantly plant-based dietary pattern that allows limited amounts of animal-source foods. It emphasizes the consumption of vegetables, fruits, whole grains, and nuts; moderate intake of seafood and poultry; and restricts red meat, added sugars, and saturated fats (Supplementary Table 1) (Willett et al., 2019). Accumulating evidence from cohort studies (Ibsen et al., 2022; Knuppel, Papier, Key, & Travis, 2019; Stubbendorff et al., 2022; Xu et al., 2021) and meta-analyses (Feng, Zhang, Wang, & Peng, 2025; Liu, Shen, & Wang, 2024) indicates that adherence to the EAT–Lancet diet could reduce the risk of chronic noncommunicable diseases (such as cardiovascular disease and cancer) and all-cause mortality, highlighting its potential as a comprehensive health-promoting strategy.

To date, no randomized controlled trial (RCT) has evaluated the association between the EAT–Lancet dietary pattern and NPD outcomes. Evidence to date is therefore derived primarily from observational studies, some of which have reported statistically significant associations between higher adherence to the EAT–Lancet dietary pattern and a lower risk of specific NPDs, including depression, anxiety, and stroke (Lu et al., 2024; Sawicki et al., 2024; Sotos-Prieto et al., 2025). In contrast, several other studies have observed no statistically significant associations for similar outcomes (Knuppel, Papier, Key, & Travis, 2019; Tabatabaei et al., 2025), resulting in an overall body of evidence that remains inconclusive. These discrepancies may, in part, reflect substantial methodological heterogeneity across studies, including differences in study design, population characteristics, and geographic context, as well as variability in the assessment of EAT–Lancet adherence and the definition of NPD outcomes (Ibsen et al., 2022; Nena Karavasiloglou et al., 2023). As a result, the interpretability of individual studies is limited, highlighting the need for a systematic synthesis of the existing evidence.

To the best of our knowledge, only two meta-analyses have examined the association between the EAT–Lancet dietary pattern and neuropsychiatric disease outcomes, and both were limited to stroke (Liu, Shen, & Wang, 2024; Wang et al., 2025). Quantitative syntheses for other NPDs, including depression, anxiety, and dementia, remain unavailable. As these conditions may share common biological mechanisms, such as chronic inflammation, oxidative stress, and dysregulation of the gut–brain axis (Dziedziak et al., 2025; Marrone & Coccurello, 2019), a focus on a single disorder is likely to overlook broader associations between dietary patterns and neuropsychiatric health. Given the current lack of RCTs on the EAT–Lancet dietary pattern and neuropsychiatric outcomes, we therefore conducted a comprehensive synthesis of observational studies to examine the associations between adherence to the EAT–Lancet dietary pattern and the risk of several common NPDs, aiming to provide an integrated evidence base to inform nutritional prevention strategies.

## Methods

This systematic review and meta-analysis followed the Preferred Reporting Items for Systematic Reviews and Meta-Analyses (PRISMA) guidelines (Page et al., 2021) and the Meta-analysis of Observational Studies in Epidemiology (MOOSE) guidelines (Stroup et al., 2000). The study protocol was registered with PROSPERO (CRD420251139334). The PRISMA checklist is provided in Supplementary Method 1, and minor amendments to the initial protocol are detailed in Supplementary Method 2.

### Search strategy

A comprehensive literature search was conducted from database inception to September 4, 2025, across five databases: PubMed, Web of Science (Core Collection; Supplementary Table 2), Embase, Scopus, and ProQuest Dissertations and Theses Global. As there is no dedicated MeSH term for the EAT–Lancet diet, relevant studies were identified through titles and abstracts searches using free-text keywords including ‘EAT–Lancet’, ‘plant diet’, and ‘planetary health diet’. Reference lists of articles were also scrutinized to identify additional eligible studies. The complete search strategy and the rationale for database selection are provided in Supplementary Table 3.

### Eligibility criteria

The inclusion criteria were as follows: (1) aged ≥18 years, with no restrictions on sex, race, or geographic region; (2) assessment of adherence to the EAT–Lancet diet using a validated instrument, with adherence analyzed as a continuous score or categorical groups of high vs low adherence; (3) reported outcomes of common NPDs, including depression, anxiety, schizophrenia, bipolar disorder, stroke, dementia, and cognitive decline; and (4) observational study design, including cohort, cross-sectional, or case–control studies.

Studies were excluded if they met any of the following criteria: (1) commentaries, reviews, meta-analyses, case reports, or case series; and (2) failure to report effect estimates for the outcomes of interest, such as hazard ratios (HRs), odds ratios (ORs), or regression coefficients (β).

### Exposure and outcomes

The exposure of interest was adherence to the EAT–Lancet diet. In the included studies, participants’ dietary intake was assessed using food frequency questionnaires or 24-hour dietary recalls, and adherence was subsequently quantified using validated dietary indices. Common scoring approaches and key components of the dietary indices are summarized in Supplementary Table 4.

The primary outcomes were common NPDs, including the presence or absence of depressive disorder, anxiety disorder, schizophrenia, bipolar disorder, stroke, and dementia, etc. Diagnoses of these conditions were based on established diagnostic criteria from the Diagnostic and Statistical Manual of Mental Disorders (DSM) and the International Classification of Diseases (ICD). In addition, depressive symptoms, anxiety symptoms, and cognitive function assessed using validated instruments were also considered primary outcomes. Associations were expressed as HR or OR for binary outcomes and as standardized regression coefficients (β) for continuous outcomes. Detailed information on outcome diagnostic criteria and assessment methods is provided in Supplementary Table 5. No secondary outcomes were prespecified for this review.

### Study selection and data extraction

All studies were imported into EndNote 21 (Clarivate, Philadelphia, PA, USA), and duplicates were removed. Two authors (Y.W. and Z.L.) independently screened titles and abstracts, followed by full texts, according to predefined inclusion and exclusion criteria. Discrepancies were resolved through discussion or consultation with a third author (Y.J.) if consensus could not be reached. Extracted information included study characteristics (first author, publication year, country), participant details (age range or mean/median age, sample size), methodological aspects (recruitment period, follow-up duration, study design), exposure assessment (dietary measurement or scoring system), health outcomes, number of events (cases), and adjusted effect estimates with 95% confidence intervals (95% CIs). When studies reported association estimates across multiple waves, we extracted the estimate from the authors’ primary longitudinal model (McKenzie et al., 2019). Corresponding authors were contacted for missing or incomplete data; however, no additional data were obtained.

### Quality assessment and GRADE assessment

Two reviewers (Y.W. and Z.L.) independently assessed the methodological quality (risk of bias) of each included study and rated the overall certainty of evidence for the main outcomes. Risk of bias at the individual study level was assessed using design-specific instruments, while the certainty of evidence for each outcome was rated using a structured grading approach. Any disagreements were resolved through discussion.

The quality of cohort studies was assessed using the Newcastle-Ottawa Scale (NOS), which comprises three domains: participant selection (4 points), comparability of groups (2 points), and outcome ascertainment (3 points). Total scores range from 0 to 9, with 7–9 indicating high quality, 4–6 moderate quality, and <4 low quality (Wells et al., 2000) (Supplementary Method 3). Cross-sectional studies were evaluated using the 11-item Agency for Healthcare Research and Quality (AHRQ) checklist, excluding one inapplicable item. Total scores range from 0 to 10, with 8–10 rated good (8–10), 4–7 fair, and 0–3 poor (Pequeno et al., 2020) (Supplementary Method 4). Evidence quality for main outcomes was assessed using the Grading of Recommendations, Assessment, Development and Evaluation (GRADE) approach, which evaluates the risk of bias, inconsistency, indirectness, imprecision, and publication bias (Guyatt et al., 2008).

### Data synthesis and analysis

Effect estimates for binary outcomes were pooled on the logarithmic scale and subsequently back-transformed to obtain combined effect sizes (Deeks, Higgins, Altman, & Group, 2019). We prioritized studies that reported adherence to the EAT–Lancet dietary pattern as a dichotomous exposure, defined as high vs low adherence, with associations expressed as HR or OR. For dementia, however, pooled estimates were derived from studies reporting a one-unit increase in adherence score, owing to the absence of data using categorical exposure measures. For anxiety and depression, the majority of cross-sectional studies reported OR, whereas only one cohort study provided HR (Lu et al., 2024). Given the low incidence of these outcomes, defined as less than ten percent, HR was approximated as OR to enable pooled analyses (Dumas & Stensrud, 2025; Liu, Shen, & Wang, 2024). Outcomes for which quantitative synthesis was not feasible were summarized narratively.

All analyses were performed using Stata version 16 (StataCorp, College Station, TX, USA). Random-effects models were applied when heterogeneity was high (*P* < 0.10 or *I* ^2^ > 50%), otherwise, fixed-effects models were used (Deeks, Higgins, Altman, & Group, 2019; Higgins, Thompson, Deeks, & Altman, 2003). Heterogeneity across studies was assessed using Cochran’s *Q* test and the *I* ^2^ statistic, and potential sources of heterogeneity were further explored using Galbraith plots (Galbraith, 1988; StataCorp, 2021). Influence analyses (a type of sensitivity analysis) were conducted to evaluate the impact of individual studies on pooled estimates. Where multiple publications originated from shared cohorts, such as the UK Biobank or the National Health and Nutrition Examination Survey (NHANES), sensitivity analyses excluding overlapping studies were performed to assess the robustness of the pooled estimates. Publication bias was assessed using Egger’s test.

Subgroup analyses were conducted to investigate potential sources of heterogeneity and differences in effect estimates. Analyses were stratified by study design, type of outcome data, dietary scoring system, statistical model, and specific NPD subtypes or cognitive domains.

## Results

### Search results and study characteristics

**Figure 1.**
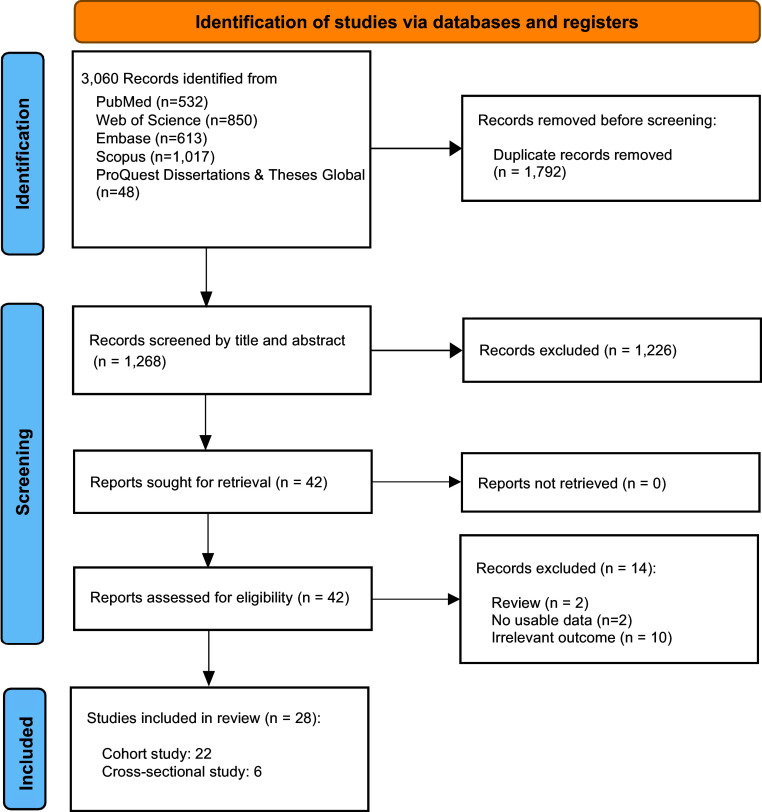
Flow diagram of the eligibility process. Figure 1. long description.

The literature search yielded 3,060 records, of which 1,792 duplicates were removed. After title and abstract screening, 1,226 records were excluded as irrelevant. During full-text review, 14 studies were further excluded, including two reviews, two studies with unusable data, and ten studies with irrelevant outcomes. Eventually, 28 studies met the inclusion criteria, comprising 22 cohort studies and 6 cross-sectional studies (Figure 1). The included reports yielded a total analytic sample of 2,211,694 participants, with a minimum of 1,200,069 unique participants after de-duplication of overlapping cohorts. Supplementary Table 6 summarizes the key characteristics of the included studies, including the number of studies per outcome, outcome data type, geographic region, and repeated cohorts. Detailed characteristics of each included study are presented in Table 1.

**Table 1.** Baseline characteristics of the studies included in meta-analysis Table 1. long description.

Study	Country	Time recruitment	Follow-up duration (years)	N cases, total sample size	Diet measurement, scoring system	Diet components or scoring groups	Disease (outcome)	Outcome source	Effect estimate (95% CI)	Effect type
Knuppel, Papier, Key, & Travis, 2019	British	1993–2001	up to 23.6	968/46,048	FFQ/Knuppel (0–14 scores)	14 components, Q1–Q4	Stroke	HRNR	1.07 (0.88, 1.30)	HR
Ibsen et al., 2022	Danes	1993–1997	15 (median)	2,253/55,016	FFQ/Knuppel (0–14 scores)	14 components, Q1–Q5	Stroke	HRNR	0.91 (0.76, 1.09)	HR
				1,858/55,016			Ischemic stroke		0.98 (0.80, 1.19)	
				267/55,016			Hemorrhagic stroke		0.99 (0.59, 1.67)	
Berthy et al., 2022	French	2009–2021	8.1 (median)	119/68,247	FFQ/ELD (−162–332 scores)	13 components, Q1–Q5	Ischemic stroke	HRNR	1.35 (0.73, 2.51)	HR
Colizzi et al., 2023	Dutch	1993–1997	15.1 (median)	838/35,496	FFQ/Colizzi (0–140 scores)	14 components, Q1–Q4	Stroke	HRNR	0.89 (0.72, 1.10)	HR
				478/35,496			Ischemic stroke		0.90 (0.68, 1.20)	
				233/35,496			Hemorrhagic stroke		0.92 (0.61, 1.38)	
Karavasiloglou et al., 2023	British	2006–2010	12.0 (mean)	2,901/473,836	FFQ/Knuppel (0–14 scores)	14 components, Q1–Q3	Stroke	HRNR	1.03 (0.87, 1.23)	HR
				2,049/473,836			Ischemic stroke		1.04 (0.84, 1.28)	
				414/473,836			Hemorrhagic stroke		1.18 (0.76, 1.83)	
Stubbendorff et al., 2024	Danes	1993–1997	10	1,359/52,452	FFQ/Knuppel (0–14 scores)	14 components, Q1–Q4	Stroke	HRNR	0.72 (0.63, 0.81)	HR
	Swedes	1991–1996	10	694/20,973	FFQ/Knuppel (0–14 scores)	14 components, Q1–Q4	Stroke	HRNR	0.70 (0.60, 0.85)	HR
Lu et al., 2024	British	2006–2010	11.62 (median)	4,548/180,446	FFQ/Knuppel (0–14 scores)	14 components, Q1–Q4	Depression	HRNR	0.81 (0.73, 0.89)	HR
				6,026/180,446			Anxiety		0.82 (0.75, 0.89)	
Colizzi et al., 2024	European	NA	12.8 (median)	7,088/364,745	FFQ/Colizzi (0–140 scores)	14 components, Q1-Q4	Stroke	HRNR	0.59 (0.54, 0.64)	HR
Sawicki et al., 2024	American	1986–1991	24	4,566/109,805	FFQ/PHDI (0–140 scores)	15 components, Q1–Q5	Stroke	HRNR	0.86 (0.78, 0.95)	HR
				2,337/109,805			Ischemic stroke		0.86 (0.75, 0.99)	
Ye et al., 2024	British	2009–2012	9.9 (median)	1,455/114,165	FFQ/Colizzi (0–140 scores)	14 components, Q1-Q4	Stroke	HRNR	0.88 (0.75, 1.04)	HR
Zhang et al., 2024	Singapore Chinese	1993–1998	20	2,397/16,736	FFQ/Colizzi (0–140 scores)	14 components, Q1–Q5	Cognitive impairment	SM-MMSE	0.72 (0.62, 0.83)	OR
van Soest et al., 2024	Dutch	2008–2013	2	NA/630	FFQ/Stubbendorff (0–42 scores)	14 components, Q1–Q3	Cognition	Neuropsychological test battery	0.12 (0.05, 0.20)	β
				NA/630			Memory		0.08 (−0.08, 0.24)	
				NA/630			Attention and working memory		0.27 (0.10, 0.45)	
				NA/630			Information processing speed		0.06 (−0.07, 0.20)	
				NA/630			Executive functioning		0.13 (0.00, 0.26)	
Goncalves et al., 2024	Brazilian	2008–2010	10	NA/11,737	FFQ/PHDI (0–150 scores)	16 components, Q1–Q5	Memory	Neuropsychological test battery	0.002 (−0.001, 0.005)*	β
				NA/11,737			Verbal fluency		0.000 (−0.002, 0.004)*	
				NA/11,737			Executive function		−0.003 (−0.007, 0.001)*	
				NA/11,737			Cognition		0.001 (−0.001, 0.003)*	
Karavasiloglou et al., 2025	British	2006–2010	13.5–14.8	1,060/196,099	FFQ/Knuppel (0–14 scores)	14 components, Q1–Q3	Stroke	HRNR	0.98 (0.94, 1.03)	HR
				755/196,099			Ischemic stroke		0.95 (0.90, 1.01)	
				160/196,099			Hemorrhagic stroke		1.01 (0.89, 1.14)	
Sotos-Prieto et al., 2025	British	2006–2010	9.4	1,268/118,469	FFQ/PHDI (0–130 scores)	14 components, Q1–Q4	Stroke	HRNR	0.82 (0.70, 0.97)	HR
Zhao et al., 2025	British	2006–2010	12.2 (median)	1,728/190,893	FFQ/Knuppel (0–14 scores)	14 components, continuous	Dementia	HRNR	0.97 (0.93, 1.02)	HR
Samuelsson et al., 2025	Swedish	1991–1996	18 (mean)	1,783/25,898	FFQ/Knuppel (0–14 scores)	14 components, continuous	Dementia	HRNR	0.95 (0.90, 1.01)	HR
				1,040/25,898			Alzheimer’s disease		0.96 (0.89, 1.03)	
				423/25,898			Vascular dementia		0.97 (0.86, 1.09)	
Wu et al., 2025	Chinese	1989–1997	10 (mean)	404/14,652	FFQ/PHDI (0–150 scores)	16 components, Q1–Q4	Stroke	HRNR	0.82 (0.60, 1.10)	HR
Li et al., 2025	Chinese	2008	Up to 10	1,785/6,678	FFQ/PHDI (0–30 scores)	10 components, Q1–Q4	Cognitive impairment	MMSE	0.54 (0.46, 0.63)	HR
Wijnhoven, Visser, Kok, & Olthof, 2025	Dutch	1992–2013	10	NA/1,340	FFQ/Stubbendorff (0–42 scores)	14 components, Q1–Q5	Cognition	Neuropsychological test battery	0.00 (−0.01, 0.02)*	β
				NA/1,340			Cognition		0.04 (−0.10, 0.18)	
				NA/1,333			Information Processing speed		0.01 (0.00, 0.02)*	
				NA/1,335			Episodic memory		−0.01 (−0.02, 0.00)*	
				NA/1,338			Executive function		0.00 (0.00, 0.01)*	
Tang et al., 2025	Chinese	1997	12 (median)	NA/3,404	FFQ/Colizzi (0–140 scores)	14 components, Q1–Q5	Memory	Neuropsychological test battery	0.025 (0.000, 0.049)*	β
				NA/3,404			Attention		0.011 (−0.018, 0.040)*	
				NA/3,404			Calculation		0.024 (−0.001, 0.048)*	
				NA/3,404			Cognition		0.020 (0.004, 0.037)*	
Yang, Sullivan, & Rebholz, 2025	American	1987–1995	NA	13,095	NA	NA, Q1–Q5	Stroke	HRNR	0.77 (0.65, 0.92)	HR
Kamrani, Kachouei, Sobhani, & Khosravi, 2024	Iranians	2017–2020	Cross-sectional studies	NA/4,579	FFQ/PHDI (0–150 scores)	16 components, Q1–Q4	Depression	DASS–21	0.65 (0.48, 0.88)	OR
							Anxiety		0.81 (0.62, 1.07)	
Jiang, Choi, & Gong, 2025	American	2005–2018	Cross-sectional studies	2,774/30,466	FFQ/Colizzi (0–140 scores)	14 components, Q1–Q4	Depression	PHQ–9	0.73 (0.58, 0.98)	OR
Lan et al., 2025	American	2005–2018	Cross-sectional studies	2,182/25,312	FFQ/Colizzi (0–140 scores)	14 components, Q1–Q4	Depression	PHQ–9	0.69 (0.55, 0.86)	OR
Tabatabaei et al., 2025	Iranian	2018–2019	Cross-sectional studies	NA/1,994	FFQ/ELD (−160.44–105.63 scores)	14 components, Q1–Q3	Anxiety	HADS	0.81 (0.61, 1.09)	OR
			Cross-sectional studies				Depression		0.78 (0.57, 1.05)	
Tan et al., 2025	American	2005–2018	Cross-sectional studies	NA/27,868	24hR/PHDI-US (0–150 scores)	16 components, Q1–Q4	Depression	PHQ–9	0.72 (0.62, 0.83)	OR
Samuelsson et al., 2025	Swedish	2014–2016	Cross-sectional studies	NA/615	Interview/Stubbendorff (0–42 scores)	14 components, continuous	Cognition	Neuropsychological test battery	0.035 (0.011,0.060)	β

*Note:* FFQ, ‘food frequency questionnaire’; 24hR, 24-hour dietary recall; PHDI, ‘planetary health diet index’; PHDI-US, ‘planetary health diet index adapted for the U.S’. population; ELD, ‘EAT–Lancet diet index’; Q1–Q5/Q1–Q4/Q1–Q3, Quantile groups (from lowest to highest adherence score quartiles or quintiles); NA, ‘not available’; N Cases, Number of cases; HRNR, ‘hospital records & national registries’; (SM-)MMSE, ‘(Singapore-modified) mini-mental state examination’, poor cognitive function was defined using education-specific cut-offs: <18 (no formal education), <21 (primary), and <25 (secondary or higher); DASS-21, ‘depression, anxiety and stress scale–21 items’; PHQ-9, ‘patient health questionnaire-9’, 0–27 (depression: ≥10); HADS, ‘hospital anxiety and depression scale’, 0–21 per subscale (normal ≤7; case ≥8); CI, ‘confidence interval’; HR, ‘hazards ratio’; OR, ‘odds ratio’; β, regression coefficient; * indicates that the analysis considered the interaction with age. The EAT–Lancet diet adherence scores labeled with the names of researchers (e.g. Knuppel, Colizzi, Stubbendorff) refer to different versions of dietary indices developed by these investigators, each based on the EAT–Lancet reference diet but with slight variations in scoring methods and operational definitions.

### EAT–Lancet diet and emotion

**Figure 2.**
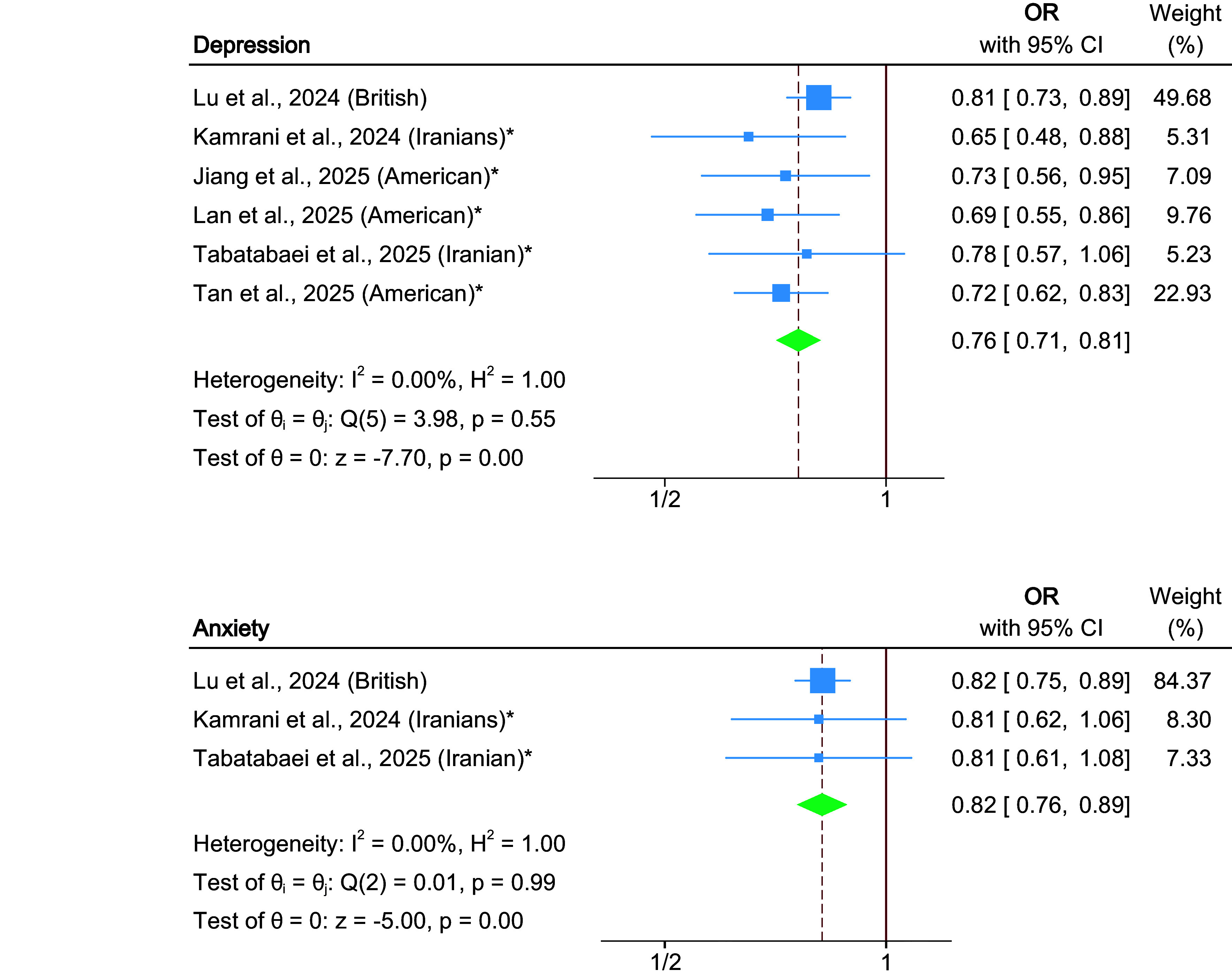
Forest plot of the association between adherence to the EAT–Lancet diet and depression and anxiety. The solid vertical line indicates the null value (OR = 1), and the dashed vertical line indicates the pooled effect estimate for the corresponding outcome. *Note:* * indicates cross-sectional studies. Figure 2. long description.

Higher adherence to the EAT–Lancet diet was associated with lower odds of depression (six studies; pooled OR, 0.76; 95% CI, 0.71–0.81; *P* < 0.001; *I* ^2^ = 0%) (Jiang, Choi, & Gong, 2025; Kamrani, Kachouei, Sobhani, & Khosravi, 2024; Lan et al., 2025; Lu et al., 2024; Tabatabaei et al., 2025; Tan et al., 2025) and anxiety (three studies; pooled OR, 0.82; 95% CI, 0.76–0.89; *P* < 0.001; *I* ^2^ = 0%) (Kamrani, Kachouei, Sobhani, & Khosravi, 2024; Lu et al., 2024; Tabatabaei et al., 2025) (Figure 2). Subgroup analyses by study design yielded consistent results. Among cross-sectional studies, EAT–Lancet diet adherence was associated with lower odds of depression (five cross-sectional studies; pooled OR, 0.71; 95% CI, 0.65–0.79; *P* < 0.001) (Jiang, Choi, & Gong, 2025; Kamrani, Kachouei, Sobhani, & Khosravi, 2024; Lan et al., 2025; Tabatabaei et al., 2025; Tan et al., 2025) and anxiety (two cross-sectional studies; pooled OR, 0.81; 95% CI, 0.66–0.99; *P* = 0.038) (Kamrani, Kachouei, Sobhani, & Khosravi, 2024; Tabatabaei et al., 2025). Similarly, the cohort study (Lu et al., 2024) reported reduced risk of depression (one study; HR, 0.81; 95% CI, 0.73–0.89; *P* < 0.001) and anxiety (one study; HR, 0.82; 95% CI, 0.75–0.89; *P* < 0.001) (Table 2 and Supplementary Figure 1).

**Table 2.** Meta-analysis for subgroup analyses Table 2. long description.

Health outcome	Subgroup	Number of studies	Overall result (95% CI)	Test of overall effect (*p*-value)
Stroke	Ischemic stroke	6	HR: 0.95 (0.90, 0.99)	*p* = 0.020
	Hemorrhagic stroke	4	HR: 1.01 (0.91, 1.13)	*p* = 0.830
	Knuppel score (total stroke)	5	HR: 0.89 (0.77, 1.03)	*p* = 0.110
	Other scores (total stroke)	7	HR: 0.79 (0.70, 0.89)	*p* < 0.001
Depression	Cross-sectional studies (Symptoms)	5	OR: 0.71 (0.65, 0.79)	*p* < 0.001
	Cohort study (diagnosis)	1	HR: 0.81 (0.73, 0.89)	*p* < 0.001
Anxiety	Cross-sectional studies (Symptoms)	2	OR: 0.81 (0.67, 0.98)	*p* = 0.038
	Cohort study (diagnosis)	1	HR: 0.82 (0.75, 0.89)	*p* < 0.001
Cognition	Change*	3	β: 0.00 (−0.00, 0.01)	*p* = 0.337
	Level*	2	β: 0.10 (0.04, 0.17)	*p* = 0.002
	Memory	3	β: −0.00 (−0.01, 0.01)	*p* = 0.660
	Attention	2	β: 0.13 (−0.13, 0.38)	*p* = 0.329
	Executive function	3	β: −0.00 (−0.00, 0.00)	*p* = 0.271
	Information processing speed	2	β: 0.01 (0.00, 0.02)	*p* = 0.043

*Note:* *Level model: examines the association between diet and cognitive function or simple change over time, without including an interaction with age; Change model: association between diet and cognitive function considering the interaction with age. For depression and anxiety, outcome type (symptom-based vs diagnosis-based) corresponded directly to study design: cross-sectional studies assessed symptoms, whereas cohort studies evaluated clinical diagnoses.

Egger’s tests did not indicate evidence of publication bias for depression (*P* = 0.157) or anxiety (*P* = 0.912) (Supplementary Table 7). Galbraith plots showed low heterogeneity for both outcomes, aligning with *I* ^2^ values of 0% (Supplementary Figure 2A, 2B).

### EAT–Lancet diet and stroke

**Figure 3.**
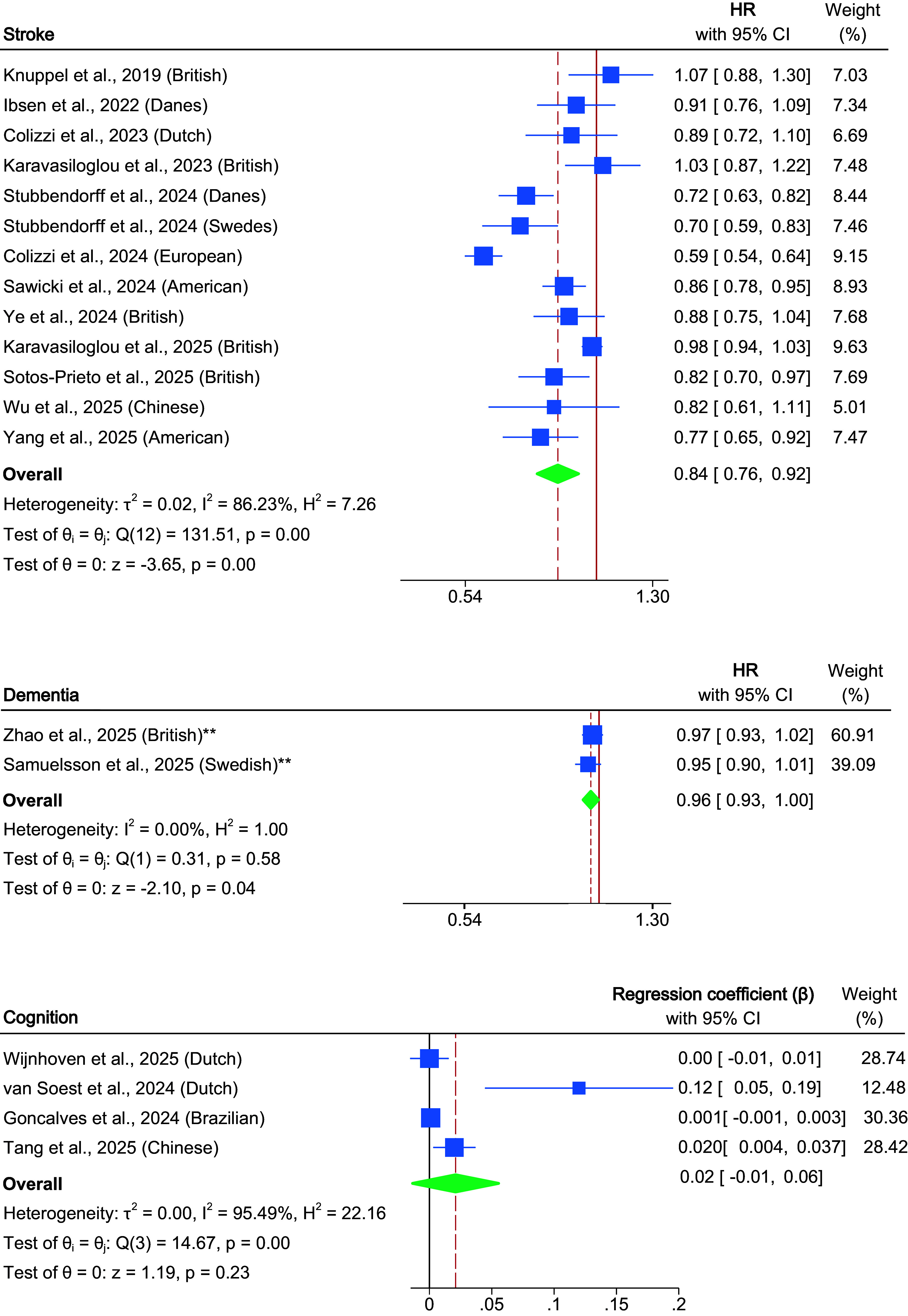
Forest plot of the association between adherence to the EAT–Lancet diet and stroke, dementia, and cognitive function. The solid vertical line indicates the null value (HR = 1 for stroke and dementia; β = 0 for cognitive function), and the dashed vertical line indicates the pooled effect estimate for the corresponding outcome. Stubbendorff et al. (2024) reported cohort-specific results for Danes and Swedes without an overall combined estimate; thus, both are shown separately in this figure, while the publication is counted once in the total number of studies. *Note:* ** indicates analyses with continuous exposure variables. Figure 3. long description.

Higher adherence to the EAT–Lancet diet was associated with a reduced risk of stroke (12 studies; pooled HR, 0.84; 95% CI, 0.76–0.92; *P* < 0.001; *I* ^2^ = 86.23%) (Chiara Colizzi et al., 2024; C. Colizzi et al., 2023; Ibsen et al., 2022; N. Karavasiloglou et al., 2025; Nena Karavasiloglou et al., 2023; Knuppel, Papier, Key, & Travis, 2019; Sawicki et al., 2024; Sotos-Prieto et al., 2025; Stubbendorff et al., 2024; M. Wu et al., 2025; Yang, Sullivan, & Rebholz, 2025; Ye et al., 2024) (Figure 3). Subgroup analyses by stroke subtype revealed a significant association with ischemic stroke (six studies; pooled HR, 0.95; 95% CI, 0.90–0.99; *P* = 0.02) (Berthy et al., 2022; Colizzi et al., 2023; Ibsen et al., 2022; Karavasiloglou et al., 2025; Nena Karavasiloglou et al., 2023; Sawicki et al., 2024), but no significant association with hemorrhagic stroke (4 studies; pooled HR, 1.01; 95% CI, 0.91–1.13; *P* = 0.83) (Colizzi et al., 2023; Ibsen et al., 2022; N. Karavasiloglou et al., 2025; Nena Karavasiloglou et al., 2023). When stratified by dietary scoring system, studies applying the Knuppel score showed no significant findings (five studies; pooled HR, 0.89; 95% CI, 0.77–1.03; *P* = 0.11) (Ibsen et al., 2022; Karavasiloglou et al., 2025; Karavasiloglou et al., 2023; Knuppel, Papier, Key, & Travis, 2019; Stubbendorff et al., 2024), whereas other scoring systems demonstrated a significant inverse association (seven studies; pooled HR, 0.79; 95% CI, 0.70–0.89; *P* < 0.001) (Colizzi et al., 2024; Colizzi et al., 2023; Sawicki et al., 2024; Sotos-Prieto et al., 2025; Wu et al., 2025; Yang, Sullivan, & Rebholz, 2025; Ye et al., 2024) (Table 2 and Supplementary Figure 1).

Egger’s test did not suggest evidence of publication bias for stroke (*P* = 0.578) (Supplementary Table 7). Galbraith plots indicated the presence of heterogeneity, with one study lying outside the 95% confidence limits (Supplementary Figure 2C).

### EAT–Lancet diet, dementia, and cognition

Adherence to the EAT–Lancet diet was modestly but significantly associated with a reduced risk of dementia (two studies; pooled HR, 0.96; 95% CI, 0.93–1.00; *P* = 0.04; *I* ^2^ = 0%) (Samuelsson et al., 2025; Zhao et al., 2025). In contrast, no significant association was found for cognitive function scores (four studies; pooled β, 0.02; 95% CI, −0.01–0.06; *P* = 0.23; *I* ^2^ = 95.5%) (Figure 3). Subgroup analyses of cognitive function revealed no significant association in the Change model, which accounted for diet-age interactions (three studies; pooled β, 0.00; 95% CI, −0.01 to 0.02; *P* = 0.34) (Goncalves et al., 2024; Tang et al., 2025; Wijnhoven, Visser, Kok, & Olthof, 2025). In contrast, the Level model, which included age as a covariate, showed a significant positive association between the diet and cognitive function (two studies; pooled β, 0.10; 95% CI, 0.04–0.17; *P* = 0.002) (van Soest, van de Rest, Witkamp, & de Groot, 2024; Wijnhoven, Visser, Kok, & Olthof, 2025). Subdomain analyses revealed no significant associations with memory (three studies; pooled β, −0.00; 95% CI, −0.01 to 0.01; *P* = 0.05) (Goncalves et al., 2024; van Soest, van de Rest, Witkamp, & de Groot, 2024; Wijnhoven, Visser, Kok, & Olthof, 2025), attention (two studies; pooled β, 0.13; 95% CI, −0.13 to 0.38; *P* = 0.33) (Tang et al., 2025; van Soest, van de Rest, Witkamp, & de Groot, 2024), or executive function (three studies; pooled β, −0.00; 95% CI, −0.00 to 0.00; *P* = 0.27) (Goncalves et al., 2024; van Soest, van de Rest, Witkamp, & de Groot, 2024; Wijnhoven, Visser, Kok, & Olthof, 2025). However, a significant association was observed in information processing speed (two studies; pooled β, 0.01; 95% CI, 0.00–0.02; *P* = 0.04) (van Soest, van de Rest, Witkamp, & de Groot, 2024; Wijnhoven, Visser, Kok, & Olthof, 2025) (Table 2 and Supplementary Figure 1).

Egger’s test did not provide evidence of publication bias for dementia (*P* = 0.580). In contrast, Egger’s test suggested potential publication bias for cognitive function (*P* = 0.0051), which should be interpreted cautiously given the small number of included studies (*k* = 4) (Supplementary Table 7). Galbraith plots showed that all data points for dementia fell within the 95% confidence limits, with one point approaching the boundary for cognitive function, indicating heterogeneity (Supplementary Figure 2D, 2E).

### Narrative synthesis

Several outcomes could not be quantitatively synthesized because of substantial methodological heterogeneity and the limited number of available studies. One study examined dementia subtypes and reported no significant associations for Alzheimer’s disease (HR, 0.96; 95% CI, 0.89–1.03) and vascular dementia (HR, 0.97; 95% CI, 0.86–1.09) (Sawicki et al., 2024). Two studies assessing cognitive impairment reported effect estimates that were not directly comparable. One study reported a hazard ratio (HR, 0.54; 95% CI, 0.46–0.63) (Li et al., 2025), whereas the other reported an odds ratio (OR, 0.72; 95% CI, 0.62–0.83) (Zhang et al., 2024). Conversion between these measures was not feasible due to high absolute risk (≥ 10%) and unavailable incidence rates. An exploratory combination of the two estimates yielded a pooled effect of 0.63 (95% CI, 0.57–0.70; *P* < 0.001) (Supplementary Figure 3); however, this result should be interpreted with caution given the incompatibility of effect measures and the inability to harmonize them. In addition, one study defined adherence to the EAT–Lancet dietary pattern as a 10% increment in dietary score, which differed from the exposure definitions used in other studies and therefore precluded quantitative pooling. Nevertheless, this study reported a significant positive association between adherence to the EAT–Lancet diet and cognitive function (β, 0.036; 95% CI, 0.011–0.060) (Samuelsson et al., 2025).

### Quality assessment and GRADE assessment

Among the 22 included cohort studies, 17 were rated as high quality, 2 as moderate quality, and 3 could not be quality-rated because only abstracts were available (Supplementary Table 8). All six included cross-sectional studies were rated as good quality (Supplementary Table 9). According to the GRADE assessment, the certainty of evidence was rated as very low for anxiety, depression, and cognition, and low for stroke and dementia (Supplementary Table 10). The low certainty ratings were mainly due to the observational nature of the evidence for all five outcomes and suspected publication bias for cognition.

### Sensitivity analyses

Sensitivity analyses (Supplementary Figure 4) showed that the exclusion of individual studies did not materially change the pooled estimates for anxiety, depression, or stroke, supporting the robustness of these findings. For dementia, which was informed by only two studies, a leave-one-out sensitivity analysis resulted in a non-significant association, indicating that this finding should be interpreted with caution. Four studies on stroke were derived from the UK Biobank (N. Karavasiloglou et al., 2025; Nena Karavasiloglou et al., 2023; Sotos-Prieto et al., 2025; Ye et al., 2024), three studies on depression used data from the NHANES (Jiang, Choi, & Gong, 2025; Lan et al., 2025; J.-x. Tan et al., 2025). To address potential overlap between studies based on shared cohorts, additional sensitivity analyses were conducted by retaining only one study from each cohort at a time while excluding the others. The pooled estimates obtained from these analyses were consistent with the original results, with depression ORs ranging from 0.77 to 0.79 (95% CI, 0.71–0.85) and stroke HRs ranging from 0.80 to 0.82 (95% CI, 0.71–0.92) (Supplementary Figure 5). These findings indicate that the inclusion of studies derived from overlapping cohorts had minimal impact on the overall results.

## Discussion

This study represents the first comprehensive meta-analysis to provide clinical evidence on the potential association between adherence to the EAT–Lancet dietary pattern and the risk of common NPDs. By synthesizing data from large and diverse populations across 22 cohort studies and 6 cross-sectional studies, we found that higher adherence to the EAT–Lancet diet was significantly associated with 24% lower odds of depression, 18% lower odds of anxiety, and a 16% reduced risk of stroke. In addition, a modest but statistically significant 4% reduction in the incidence of dementia was observed, whereas no significant association was identified for cognitive function. Despite these findings, the overall certainty of evidence ranged from very low for anxiety, depression, and cognitive outcomes to low for stroke and dementia, suggesting that the observed associations should be interpreted cautiously.

Our findings suggest that higher adherence to the predominantly plant-forward, nutrient-rich EAT–Lancet diet is associated with lower odds of depression and anxiety. However, the evidence base for both outcomes was largely cross-sectional, which may inflate observed associations and limit causal inference. Moreover, most cross-sectional studies captured depressive and anxiety symptoms using validated scales rather than clinically diagnosed disorders. Symptom-based measures may be more sensitive to subclinical variation and therefore could yield larger effect estimates than diagnosis-based endpoints. Nonetheless, subgroup analyses by study design were directionally consistent, with cross-sectional studies showing lower odds and the single cohort study reporting reduced risks for clinically diagnosed depression and anxiety. Overall, these findings provide preliminary support for a potential protective role of EAT–Lancet adherence, while underscoring the need for additional longitudinal studies and intervention trials incorporating standardized diagnostic outcomes to clarify causality and clinical relevance.

This meta-analysis extends the evidence indicating that greater adherence to the EAT–Lancet dietary pattern is associated with a reduced risk of stroke. Our findings differ from the earlier meta-analysis by Liu, Shen, andWang (2024), which reported no statistically significant association. In contrast, Wang et al. (2025) observed a protective association for total stroke, consistent with our results. We incorporated four additional eligible studies beyond Liu et al. and six beyond Wang et al., providing a more comprehensive and updated estimate. Sensitivity analyses addressing potential cohort overlap by retaining a single report per cohort yielded materially similar results, supporting the robustness of the association. Subgroup analyses indicated heterogeneity according to the adherence scoring approach. Studies using the Knuppel score did not reach statistical significance, whereas those applying more granular scoring systems reported statistically significant protective associations. The early 14-item Knuppel tool relies on binary (0/1) scoring for each component, which may limit its ability to differentiate levels of adherence. These findings underscore the importance of more granular and harmonized adherence metrics in future research.

Subgroup analyses further indicated a modest but statistically significant protective association for ischemic stroke, whereas no significant association was observed for hemorrhagic stroke. This difference is biologically plausible. Ischemic stroke is largely related to atherosclerotic and thromboembolic processes, which may be influenced by diet through effects on blood pressure, lipid profiles, glycemic control, adiposity, and inflammation (O’Donnell et al., 2010). In contrast, hemorrhagic stroke is more closely linked to vascular rupture and bleeding susceptibility, mechanisms that may be less directly modified by dietary patterns (O’Donnell et al., 2010). The relatively small number of hemorrhagic events may also have limited statistical power to detect modest associations. Clinically, these findings suggest that the potential benefits of EAT–Lancet adherence may be most relevant for ischemic stroke prevention. Such evidence supports dietary prevention strategies among individuals at elevated ischemic risk and highlights the importance of prioritizing ischemic stroke endpoints in future prospective studies and RCTs.

Although no significant association was observed between adherence to the EAT–Lancet diet and cognitive function scores, a significant association was observed for clinically diagnosed dementia. The pooled HR for dementia was close to 1.0, which may partly reflect the scaling of the exposure, as continuous adherence scores imply that each one-unit increase represents only a modest incremental change in adherence. Cognitive outcomes also varied substantially across studies. One possible explanation is that a single ‘global cognition’ score was derived from multiple neuropsychological tests using heterogeneous construction methods and test components. Egger’s test suggested potential publication bias in the cognition analyses, and the relatively small number of available studies further limits confidence in these findings. In addition, two studies (Li et al., 2025; Zhang et al., 2024) reported significant inverse associations with cognitive impairment, but pooling was not feasible because effect measures were not comparable; thus, these results should be interpreted as supportive rather than conclusive. Overall, the evidence for cognition remains limited and of low certainty. Adequately powered prospective studies using standardized cognitive assessments, harmonized effect measures, and longer follow-up are needed to clarify whether adherence to the EAT–Lancet dietary pattern is associated with cognitive trajectories and dementia risk.

Across depression, anxiety, stroke, and dementia, inverse associations with EAT–Lancet adherence may reflect shared biological mechanisms supporting neuropsychiatric and vascular health. As a predominantly plant-based dietary pattern, EAT–Lancet adherence may attenuate chronic low-grade inflammation and oxidative stress, processes implicated in affect regulation and vascular injury (Correia, Cardoso, & Vale, 2023; Lane et al., 2022; van Zonneveld et al., 2024; Wu et al., 2023). Higher fiber intake and greater plant diversity may also modulate the gut microbiome and gut–brain signaling, with potential downstream effects on stress responsivity, mood, and cognitive function (Berding et al., 2021; Clerici, Bottari, & Bottari, 2025). For stroke, potential benefits of EAT–Lancet adherence may additionally operate through vascular and metabolic pathways, including improved endothelial function, reduced platelet aggregation, slower atherosclerosis progression, and enhanced glucose homeostasis and insulin sensitivity. These mechanisms are central to stroke pathogenesis and may also contribute to vascular cognitive impairment (Aleksandrova, Koelman, & Rodrigues, 2021; de Oliveira Neta et al., 2024; Lin, Wang, & Huang, 2023; Meital et al., 2019; Willey et al., 2009; Zehr & Walker, 2018). While biologically plausible, these proposed mechanisms remain inferential and warrant confirmation in adequately powered prospective studies and RCTs incorporating pathway-specific biomarkers.

Our findings are broadly consistent with evidence for other plant-focused dietary patterns, including the Dietary Approaches to Stop Hypertension (DASH) diet and the Mediterranean-DASH Intervention for Neurodegenerative Delay (MIND) diet (Chen et al., 2023; J. Tan, Wang, & Tomiyama, 2023). Among these, the Mediterranean diet has the most mature evidence base, collectively suggesting that plant-based dietary patterns may confer favorable neuropsychiatric and vascular effects. Recent meta-analyses have linked higher Mediterranean diet adherence to lower risks of stroke (Ungvari et al., 2025), depression (Shafiei, Salari-Moghaddam, Larijani, & Esmaillzadeh, 2023), and dementia( Fekete et al., 2025), and RCTs have demonstrated benefits for cognitive outcomes and mood (Bayes, Schloss, & Sibbritt, 2022; Valls-Pedret et al., 2015). However, few studies have directly compared the effects of different dietary patterns on neuropsychiatric outcomes within the same populations. Comparative prospective studies are needed to determine whether the EAT–Lancet diet confers incremental or broadly comparable benefits relative to other plant-focused dietary patterns.

## Strengths and limitations

This meta-analysis represents the first comprehensive synthesis of evidence examining adherence to the EAT–Lancet diet in relation to common neuropsychiatric outcomes, suggesting that this predominantly plant-based dietary pattern may be associated with a lower risk of certain NPDs. Based on 28 studies including more than one million participants, our findings highlight diet as a potentially modifiable factor that can be addressed through both individual-level behavioral strategies and population-level interventions, supporting the EAT–Lancet diet as a promising approach to promote neurological and mental health.

Several limitations should be acknowledged. First, the inclusion of cross-sectional studies limits causal inference; however, the consistent findings from subgroup analyses across different study designs strengthen the reliability of the observed associations. Second, variations in dietary scoring systems and potential measurement errors in food frequency questionnaires (FFQs) may have introduced bias. Third, evidence of publication bias was observed for cognitive function. Fourth, the limited number of studies for specific outcomes and substantial heterogeneity in some analyses warrant cautious interpretation. Finally, potential overlap among large cohorts could have slightly inflated precision; yet, effect estimates remained largely consistent when overlapping studies were excluded.

## Conclusion

Higher adherence to the EAT–Lancet diet may be associated with lower risks of anxiety, depression, stroke, and dementia, whereas evidence for cognitive outcomes remains limited and inconsistent. Taken together, these findings suggest that the EAT–Lancet diet may represent a clinically relevant and modifiable factor with potential roles in prevention and as an adjunct to care in neuropsychiatric health. However, the results should be interpreted cautiously, given the low to very low certainty of evidence for several outcomes. Larger prospective cohort studies with repeated dietary assessments and standardized, clinically diagnosed outcomes are needed to strengthen the longitudinal evidence base. In parallel, RCTs are required to evaluate the effectiveness of EAT–Lancet-based interventions and thereby establish their clinical and preventive value.

## Supporting information

10.1017/S0033291726104528.sm001 Wang et al. supplementary material Wang et al. supplementary material

## Data Availability

All data generated or analyzed in this study are included in this article and its supplementary material files. Further inquiries may be directed to the corresponding author.

## Supplementary material

The supplementary material for this article can be found at http://doi.org/10.1017/S0033291726104528.

## Figure 1. Long description

At the top, a yellow box states ‘Identification of studies via databases and registers.’ Below, a box lists 3,060 records identified from PubMed 532, Web of Science 850, Embase 613, Scopus 1,017, ProQuest Dissertations and Theses Global 48. To the right, an arrow leads to a box: ‘Records removed before screening: Duplicate records removed 1,792.’ Downward, a box reads ‘Records screened by title and abstract 1,268.’ To the right, an arrow leads to ‘Records excluded 1,226.’ Downward, a box reads ‘Reports sought for retrieval 42.’ To the right, an arrow leads to ‘Reports not retrieved 0.’ Downward, a box reads ‘Reports assessed for eligibility 42.’ To the right, an arrow leads to ‘Records excluded 14: Review 2, No usable data 2, Irrelevant outcome 10.’ At the bottom, a box reads ‘Studies included in review 28: Cohort study 22, Cross-sectional study 6.’

Navigate back to Figure 1..

## Table 1. Long description

Column headers from left to right are Study, Country, Time recruitment, Follow-up duration in years, N cases and total sample size, Diet measurement and scoring system, Diet components or scoring groups, Disease outcome, Outcome source, Effect estimate with 95 percent confidence interval, and Effect type. Each row details a study, with some studies spanning multiple rows for different outcomes. For example, Knuppel, Papier, Key, and Travis 2019 (British, 1993–2001, up to 23.6 years, 968 cases out of 46,048) used FFQ/Knuppel scoring, 14 components in quartiles, outcome Stroke, source HRNR, effect estimate 1.07 (0.88, 1.30), effect type HR. Ibsen et al. 2022 (Danes, 1993–1997, 15 years median, 2,253/55,016) used FFQ/Knuppel, 14 components in quintiles, outcomes include Stroke 0.91 (0.76, 1.09), Ischemic stroke 0.98 (0.80, 1.19), Hemorrhagic stroke 0.99 (0.59, 1.67), all HR. Berthy et al. 2022 (French, 2009–2021, 8.1 years median, 119/68,247) used FFQ/ELD, 13 components, Ischemic stroke 1.35 (0.73, 2.51) HR. Colizzi et al. 2023 (Dutch, 1993–1997, 15.1 years median, 838/35,496) used FFQ/Colizzi, 14 components, outcomes Stroke 0.89 (0.72, 1.10), Ischemic stroke 0.90 (0.68, 1.20), Hemorrhagic stroke 0.92 (0.61, 1.38), all HR. Karavasiloglou et al. 2023 (British, 2006–2010, 12 years mean, 2,901/473,836) used FFQ/Knuppel, 14 components, outcomes Stroke 1.03 (0.87, 1.23), Ischemic stroke 1.04 (0.84, 1.28), Hemorrhagic stroke 1.18 (0.76, 1.83), all HR. Stubbendorff et al. 2024 (Danes and Swedes, 1993–1997 and 1991–1996, 10 years, 1,359/52,452 and 694/20,973) used FFQ/Knuppel, 14 components, Stroke 0.72 (0.63, 0.81) and 0.70 (0.60, 0.85) HR. Lu et al. 2024 (British, 2006–2010, 11.62 years median, 4,548/180,446 and 6,026/180,446) used FFQ/Knuppel, 14 components, Depression 0.81 (0.73, 0.89), Anxiety 0.82 (0.75, 0.89) HR. Colizzi et al. 2024 (European, NA, 12.8 years median, 7,088/364,745) used FFQ/Colizzi, 14 components, Stroke 0.59 (0.54, 0.64) HR. Sawicki et al. 2024 (American, 1986–1991, 24 years, 4,566/109,805 and 2,337/109,805) used FFQ/PHDI, 15 components, Stroke 0.86 (0.78, 0.95), Ischemic stroke 0.86 (0.75, 0.99) HR. Ye et al. 2024 (British, 2009–2012, 9.9 years median, 1,455/114,165) used FFQ/Colizzi, 14 components, Stroke 0.88 (0.75, 1.04) HR. Zhang et al. 2024 (Singapore Chinese, 1993–1998, 20 years, 2,397/16,736) used FFQ/Colizzi, 14 components, Cognitive impairment 0.72 (0.62, 0.83) OR. van Soest et al. 2024 (Dutch, 2008–2013, 2 years, NA/630) used FFQ/Stubbendorff, 14 components, outcomes Cognition 0.12 (0.05, 0.20), Memory 0.08 (–0.08, 0.24), Attention and working memory 0.27 (0.10, 0.45), Information processing speed 0.06 (–0.07, 0.20), Executive functioning 0.13 (0.00, 0.26), all beta. Goncalves et al. 2024 (Brazilian, 2008–2010, 10 years, NA/11,737) used FFQ/PHDI, 16 components, outcomes Memory 0.002 (–0.001, 0.005), Verbal fluency 0.000 (–0.002, 0.004), Executive function –0.003 (–0.007, 0.001), Cognition 0.001 (–0.001, 0.003), all beta. Karavasiloglou et al. 2025 (British, 2006–2010, 13.5–14.8 years, 1,060/196,099) used FFQ/Knuppel, 14 components, outcomes Stroke 0.98 (0.94, 1.03), Ischemic stroke 0.95 (0.90, 1.01), Hemorrhagic stroke 1.01 (0.89, 1.14), all HR. Sotos-Prieto et al. 2025 (British, 2006–2010, 9.4 years, 1,268/118,469) used FFQ/PHDI, 14 components, Stroke 0.82 (0.70, 0.97) HR. Zhao et al. 2025 (British, 2006–2010, 12.2 years median, 1,728/190,893) used FFQ/Knuppel, 14 components, Dementia 0.97 (0.93, 1.02) HR. Samuelsson et al. 2025 (Swedish, 1991–1996, 18 years mean, 1,783/25,898) used FFQ/Knuppel, 14 components, Dementia 0.95 (0.90, 1.01), Alzheimer’s disease 0.96 (0.89, 1.03), Vascular dementia 0.97 (0.86, 1.09), all HR. Wu et al. 2025 (Chinese, 1989–1997, 10 years mean, 404/14,652) used FFQ/PHDI, 16 components, Stroke 0.82 (0.60, 1.10) HR. Li et al. 2025 (Chinese, 2008, up to 10 years, 1,785/6,678) used FFQ/PHDI, 10 components, Cognitive impairment 0.54 (0.46, 0.63) HR. Wijnhoven, Visser, Kok, and Olthof 2025 (Dutch, 1992–2013, 10 years, NA/1,340) used FFQ/Stubbendorff, 14 components, outcomes Cognition 0.00 (–0.01, 0.02), Cognition 0.04 (–0.10, 0.18), Information processing speed 0.01 (0.00, 0.02), Episodic memory –0.01 (–0.02, 0.00), Executive function 0.00 (0.00, 0.01), all beta. Tang et al. 2025 (Chinese, 1997, 12 years median, NA/3,404) used FFQ/Colizzi, 14 components, outcomes Memory 0.025 (0.000, 0.049), Attention 0.011 (–0.018, 0.040), Calculation 0.024 (–0.001, 0.048), Cognition 0.020 (0.004, 0.037), all beta. Yang, Sullivan, and Rebholz 2025 (American, 1987–1995, NA, 13,095) used NA, NA, Stroke 0.77 (0.65, 0.92) HR. Kamrani, Kachouei, Sobhani, and Khosravi 2024 (Iranians, 2017–2020, cross-sectional, NA/4,579) used FFQ/PHDI, 16 components, Depression 0.65 (0.48, 0.88), Anxiety 0.81 (0.62, 1.07) OR. Jiang, Choi, and Gong 2025 (American, 2005–2018, cross-sectional, 2,774/30,466) used FFQ/Colizzi, 14 components, Depression 0.73 (0.58, 0.98) OR. Lan et al. 2025 (American, 2005–2018, cross-sectional, 2,182/25,312) used FFQ/Colizzi, 14 components, Depression 0.69 (0.55, 0.86) OR. Tabatabaei et al. 2025 (Iranian, 2018–2019, cross-sectional, NA/1,994) used FFQ/ELD, 14 components, Anxiety 0.81 (0.61, 1.09), Depression 0.78 (0.57, 1.05) OR. Tan et al. 2025 (American, 2005–2018, cross-sectional, NA/27,868) used 24hR/PHDI-US, 16 components, Depression 0.72 (0.62, 0.83) OR. Samuelsson et al. 2025 (Swedish, 2014–2016, cross-sectional, NA/615) used Interview/Stubbendorff, 14 components, Cognition 0.035 (0.011, 0.060) beta. The table footnotes define all abbreviations and clarify that different versions of the EAT–Lancet diet index are used across studies.

Navigate back to Table 1..

## Figure 2. Long description

The top panel is labeled Depression. The y-axis lists six studies: Lu et al., 2024 (British), Kamrani et al., 2024 (Iranians) with an asterisk, Jiang et al., 2025 (American) with an asterisk, Lan et al., 2025 (American) with an asterisk, Tabatabaei et al., 2025 (Iranian) with an asterisk, and Tan et al., 2025 (American) with an asterisk. The x-axis is labeled with tick marks at one half and one. Each study is represented by a blue square (size proportional to weight) and a horizontal line showing the 95 percent confidence interval. The odds ratios and confidence intervals are: Lu 0.81 [0.73, 0.89] weight 49.68 percent, Kamrani 0.65 [0.48, 0.88] weight 5.31 percent, Jiang 0.73 [0.56, 0.95] weight 7.09 percent, Lan 0.69 [0.55, 0.86] weight 9.76 percent, Tabatabaei 0.78 [0.57, 1.06] weight 5.23 percent, Tan 0.72 [0.62, 0.83] weight 22.93 percent. A green diamond at the bottom shows the pooled odds ratio 0.76 [0.71, 0.81]. Heterogeneity statistics are I squared equals 0.00 percent, H squared equals 1.00. Test of theta sub i equals theta: Q(5) equals 3.98, p equals 0.55. Test of theta equals 0: z equals negative 7.70, p equals 0.00. The bottom panel is labeled Anxiety. The y-axis lists three studies: Lu et al., 2024 (British), Kamrani et al., 2024 (Iranians) with an asterisk, Tabatabaei et al., 2025 (Iranian) with an asterisk. The odds ratios and confidence intervals are: Lu 0.82 [0.75, 0.89] weight 84.37 percent, Kamrani 0.81 [0.62, 1.06] weight 8.30 percent, Tabatabaei 0.81 [0.61, 1.08] weight 7.33 percent. The pooled odds ratio is 0.82 [0.76, 0.89]. Heterogeneity statistics are I squared equals 0.00 percent, H squared equals 1.00. Test of theta sub i equals theta: Q(2) equals 0.01, p equals 0.99. Test of theta equals 0: z equals negative 5.00, p equals 0.00. The solid vertical line at one marks the null value, and the dashed vertical line marks the pooled effect estimate for each outcome. Asterisks indicate cross-sectional studies.

Navigate back to Figure 2..

## Table 2. Long description

The table contains five columns: Health outcome, Subgroup, Number of studies, Overall result with 95 percent confidence interval, and Test of overall effect p-value. For stroke: Ischemic stroke (6 studies, H R 0.95, 0.90 to 0.99, p equals 0.020), Hemorrhagic stroke (4, H R 1.01, 0.91 to 1.13, p equals 0.830), Knuppel score total stroke (5, H R 0.89, 0.77 to 1.03, p equals 0.110), Other scores total stroke (7, H R 0.79, 0.70 to 0.89, p less than 0.001). For depression: Cross-sectional studies symptoms (5, O R 0.71, 0.65 to 0.79, p less than 0.001), Cohort study diagnosis (1, H R 0.81, 0.73 to 0.89, p less than 0.001). For anxiety: Cross-sectional studies symptoms (2, O R 0.81, 0.67 to 0.98, p equals 0.038), Cohort study diagnosis (1, H R 0.82, 0.75 to 0.89, p less than 0.001). For cognition: Change (3, beta 0.00, minus 0.00 to 0.01, p equals 0.337), Level (2, beta 0.10, 0.04 to 0.17, p equals 0.002), Memory (3, beta minus 0.00, minus 0.01 to 0.01, p equals 0.660), Attention (2, beta 0.13, minus 0.13 to 0.38, p equals 0.329), Executive function (3, beta minus 0.00, minus 0.00 to 0.00, p equals 0.271), Information processing speed (2, beta 0.01, 0.00 to 0.02, p equals 0.043). Footnote explains level and change models for cognition and outcome types for depression and anxiety.

Navigate back to Table 2..

## Figure 3. Long description

At the top is the stroke panel, listing thirteen cohort studies from various countries. The leftmost column names each study, followed by hazard ratios with 95 percent confidence intervals, then weight percentages. Blue squares represent individual study estimates, with horizontal lines indicating confidence intervals. The solid vertical line marks the null value at hazard ratio equals one, and the dashed line marks the pooled effect estimate. The overall pooled hazard ratio for stroke is 0.84 with a confidence interval of 0.76 to 0.92. Below, the dementia panel lists two studies, showing hazard ratios of 0.97 and 0.95, with the pooled estimate at 0.96 and confidence interval 0.93 to 1.00. The cognition panel at the bottom lists four studies, presenting regression coefficients with 95 percent confidence intervals and weights. The pooled regression coefficient is 0.02 with a confidence interval of minus 0.01 to 0.06. Each panel includes heterogeneity statistics and test results for effect estimates. Analyses with continuous exposure variables are marked with double asterisks.

Navigate back to Figure 3..
