# Supplementary material for: Adherence to the EAT–Lancet diet and neuropsychiatric disorders: a systematic review and meta-analysis
Source: Psychol Med. 2026 Jun 1;56:e171. doi: 10.1017/S0033291726104528 (PMC13234517; doi:10.1017/S0033291726104528)
Supplement: Wang et al. supplementary material [file S0033291726104528sup001.pdf]

Supplementary files

Supplementary Method 1. PRISMA 2020 checklist.

Supplementary Method 2. Amendments to Registration and Rationale.

Supplementary Method 3. Newcastle–Ottawa Scale (NOS) criteria for cohort studies.

Supplementary Method 4. Agency for Healthcare Research and Quality (AHRQ) 11-item checklist for cross-sectional studies.

Supplementary Table 1. Healthy reference diet, with possible ranges, for an intake of 2500 kcal/day.

Supplementary Table 2. Databases included in the Web of Science Core Collection.

Supplementary Table 3. Literature search strategy and outcomes.

Supplementary Table 4. Summary of EAT-Lancet/Planetary Health Diet adherence scoring methods.

Supplementary Table 5. Summary of outcome assessment and definitions.

Supplementary Table 6. Summary of included study designs and key characteristics.

Supplementary Table 7. Assessment of publication bias using Egger's test.

Supplementary Table 8. Quality assessment overview of the Cohort studies.

Supplementary Table 9. Quality assessment overview of the Cross-sectional studies.

Supplementary Table 10. Certainty of evidence for the associations between EAT-Lancet diet and neuropsychiatric disorders with GRADE.

Supplementary Figure 1. The forest plots of meta-analysis for subgroup analyses.

Supplementary Figure 2. The Galbraith plot of meta-analysis.

Supplementary Figure 3. The forest plots of cognitive impairment.

Supplementary Figure 4. Results of sensitivity analysis (influence analyses).

Supplementary Figure 5. Sensitivity analysis assessing the impact of overlapping publications from the same cohort on the pooled estimates.

Supplementary Method 1. PRISMA 2020 checklist

Section and Topic	Item #	Checklist item	Location where item is reported
TITLE			
Title	1	Identify the report as a systematic review.	Page 1
ABSTRACT			
Abstract	2	See the PRISMA 2020 for Abstracts checklist.	Page 3
INTRODUCTION			
Rationale	3	Describe the rationale for the review in the context of existing knowledge.	Page 4-6
Objectives	4	Provide an explicit statement of the objective(s) or question(s) the review addresses.	Page 4-6
METHODS			
Eligibility criteria	5	Specify the inclusion and exclusion criteria for the review and how studies were grouped for the syntheses.	Page 7
Information sources	6	Specify all databases, registers, websites, organisations, reference lists and other sources searched or consulted to identify studies. Specify the date when each source was last searched or consulted.	Page 6
Search strategy	7	Present the full search strategies for all databases, registers and websites, including any filters and limits used.	Supplementary Table 3
Selection process	8	Specify the methods used to decide whether a study met the inclusion criteria of the review, including how many reviewers screened each record and each report retrieved, whether they worked independently, and if applicable, details of automation tools used in the process.	Page 8
Data collection process	9	Specify the methods used to collect data from reports, including how many reviewers collected data from each report, whether they worked independently, any processes for obtaining or confirming data from study investigators, and if applicable, details of automation tools used in the process.	Page 8
Data items	10a	List and define all outcomes for which data were sought. Specify whether all results that were compatible with each outcome domain in each study were sought (e.g. for all measures, time points, analyses), and if not, the methods used to decide which results to collect.	Page 7
	10b	List and define all other variables for which data were sought (e.g. participant and intervention characteristics, funding sources). Describe any assumptions made about any missing or unclear information.	Page 7

Section and Topic	Item #	Checklist item	Location where item is reported
Study risk of bias assessment	11	Specify the methods used to assess risk of bias in the included studies, including details of the tool(s) used, how many reviewers assessed each study and whether they worked independently, and if applicable, details of automation tools used in the process.	Page 8
Effect measures	12	Specify for each outcome the effect measure(s) (e.g. risk ratio, mean difference) used in the synthesis or presentation of results.	Page 9
Synthesis methods	13a	Describe the processes used to decide which studies were eligible for each synthesis (e.g. tabulating the study intervention characteristics and comparing against the planned groups for each synthesis (item #5)).	Page 7-10
	13b	Describe any methods required to prepare the data for presentation or synthesis, such as handling of missing summary statistics, or data conversions.	Page 9-10
	13c	Describe any methods used to tabulate or visually display results of individual studies and syntheses.	Page 9-10
	13d	Describe any methods used to synthesize results and provide a rationale for the choice(s). If meta-analysis was performed, describe the model(s), method(s) to identify the presence and extent of statistical heterogeneity, and software package(s) used.	Page 9-10
	13e	Describe any methods used to explore possible causes of heterogeneity among study results (e.g. subgroup analysis, meta-regression).	Page 9-10
	13f	Describe any sensitivity analyses conducted to assess robustness of the synthesized results.	Page 9-10
Reporting bias assessment	14	Describe any methods used to assess risk of bias due to missing results in a synthesis (arising from reporting biases).	Page 8-9
Certainty assessment	15	Describe any methods used to assess certainty (or confidence) in the body of evidence for an outcome.	Page 8-9
**RESULTS**			
Study selection	16a	Describe the results of the search and selection process, from the number of records identified in the search to the number of studies included in the review, ideally using a flow diagram.	Page 10 and Figure 1
	16b	Cite studies that might appear to meet the inclusion criteria, but which were excluded, and explain why they were excluded.	Page 10 and Figure 1

Section and Topic	Item #	Checklist item	Location where item is reported
Study characteristics	17	Cite each included study and present its characteristics.	Page 10, Supplementary Table 6 and Table 1
Risk of bias in studies	18	Present assessments of risk of bias for each included study.	Supplementary Table 7
Results of individual studies	19	For all outcomes, present, for each study: (a) summary statistics for each group (where appropriate) and (b) an effect estimate and its precision (e.g. confidence/credible interval), ideally using structured tables or plots.	Page 10
Results of syntheses	20a	For each synthesis, briefly summarise the characteristics and risk of bias among contributing studies.	Page 8-10
	20b	Present results of all statistical syntheses conducted. If meta-analysis was done, present for each the summary estimate and its precision (e.g. confidence/credible interval) and measures of statistical heterogeneity. If comparing groups, describe the direction of the effect.	Figure 2-3 and Supplementary Figure 1
	20c	Present results of all investigations of possible causes of heterogeneity among study results.	Page 8-11
	20d	Present results of all sensitivity analyses conducted to assess the robustness of the synthesized results.	Page 11
Reporting biases	21	Present assessments of risk of bias due to missing results (arising from reporting biases) for each synthesis assessed.	Page 10-12, Figure 2 and 3
Certainty of evidence	22	Present assessments of certainty (or confidence) in the body of evidence for each outcome assessed.	Page 13 and Supplementary Table 10
**DISCUSSION**			
Discussion	23a	Provide a general interpretation of the results in the context of other evidence.	Page 14-16
	23b	Discuss any limitations of the evidence included in the review.	Page 18
	23c	Discuss any limitations of the review processes used.	Page 18
	23d	Discuss implications of the results for practice, policy, and future research.	Page 18-19

Section and Topic	Item #	Checklist item	Location where item is reported
**OTHER INFORMATION**			
Registration and protocol	24a	Provide registration information for the review, including register name and registration number, or state that the review was not registered.	Page 6
	24b	Indicate where the review protocol can be accessed, or state that a protocol was not prepared.	Page 6 and Supplementary Method 2
	24c	Describe and explain any amendments to information provided at registration or in the protocol.	Supplementary Method 2
Support	25	Describe sources of financial or non-financial support for the review, and the role of the funders or sponsors in the review.	Page 20
Competing interests	26	Declare any competing interests of review authors.	Page 20
Availability of data, code and other materials	27	Report which of the following are publicly available and where they can be found: template data collection forms; data extracted from included studies; data used for all analyses; analytic code; any other materials used in the review.	Page 21

From: Page MJ, McKenzie JE, Bossuyt PM, Boutron I, Hoffmann TC, Mulrow CD, et al. The PRISMA 2020 statement: an updated guideline for reporting systematic reviews. BMJ 2021;372:n71. doi: 10.1136/bmj.n71

Supplementary Method 2. Amendments to Registration and Rationale.

The registered protocol was titled: Association between EAT-Lancet Planetary Health Diet and Risk of Neuropsychiatric Disorders: A Systematic Review and Meta-analysis

PROSPERO 2025 CRD420251139334. Available from:

https://www.crd.york.ac.uk/PROSPERO/view/CRD420251139334.

1. In the registered protocol, we planned to assess publication bias visually using funnel plots and statistically using Egger's test. However, for most outcomes the number of included studies was fewer than 10, a situation in which funnel plots are generally considered uninformative and may be misleading. Therefore, we did not construct funnel plots and instead assessed publication bias using Egger's test where appropriate.
2. We incorporated Galbraith (radial) plots into the statistical analysis plan to better identify potential sources of heterogeneity across the included studies. While the Q test and the I^2 statistic quantify the extent of heterogeneity, they do not indicate which studies contribute most strongly to it. Galbraith plots provide a visual diagnostic tool for detecting outliers and influential studies, thereby improving the transparency and robustness of our meta-analysis.
3. We did not search the Cochrane Central Register of Controlled Trials (CENTRAL) as originally specified in the protocol, because CENTRAL primarily indexes randomized controlled trials, and randomized trials were not eligible for inclusion in this review.
4. Conference abstracts were not excluded. Conference abstracts were not excluded to minimize publication bias and capture the most up-to-date evidence. When sufficient methodological details and outcome data were available, they were screened and included in the qualitative synthesis and/or quantitative analyses.

Supplementary Method 3. Newcastle–Ottawa Scale (NOS) criteria for cohort studies(Wells et al., 2000).

I. Selection:

1. Representativeness of the exposed cohort (1 point)

   - Truly representative of the average community or subgroup (1 point)

   - Somewhat representative of the average community or subgroup (0.5 point)

   - Selected group of users or no description of the derivation of the cohort (0 point)

2. Selection of the non-exposed cohort (1 point)

   - Drawn from the same community as the exposed cohort and disease-free at baseline (1 point)

   - Drawn from the same community as the exposed cohort but not disease-free at baseline (0.5 point)

   - Drawn from a different source or no description of the non-exposed cohort, or potential selection bias (0 point)

3. Ascertainment of exposure (1 point maximum)

   - Secure record (e.g., biomarkers) or structured interview/professional assessment (1 point)

   - Self-report (e.g., questionnaire) (0.5 point)

   - No description (0 point)

4. Demonstration that outcome of interest was not present at start of study (Yes, 1 point)

II. Comparability:

1. Comparability of cohorts on the basis of the design or analysis (1 point)

   - Study controls for the most important confounders fully and appropriately, or study design inherently accounts for confounding (1 point)

   - Study partially controls for confounders (0.5 point)

   - No control for confounders (0 point)

2. Comparability of cohorts on the basis of the measurement (1 point)

   - Cohorts are comparable on the most important confounders, or effective adjustment has been made (1 point)

   - Cohorts differ on some confounders or adjustment is incomplete (0.5 point)

   - Cohorts differ on multiple confounders, or comparability not described (0 point)

III. Outcome:

1. Assessment of outcome (1 point)

   - Independent blind assessment (1 point)

   - Record linkage, e.g., hospital or insurance data, radiological or histological confirmation (1 point)

- Self-report (0 point)

- No description (0 point)

2. Was follow-up long enough for outcomes to occur? (Yes, 1 point)

3. Adequacy of follow-up of cohorts (1 point)

- Complete follow-up for all subjects (1 point)

- Follow-up rate >90%, with all dropouts having reasonable explanations (1 point)

- Follow-up rate <90% or reasons for loss to follow-up potentially related to outcomes of interest (0 point)

- No description (0 point)

Supplementary Method 4. Agency for Healthcare Research and Quality (AHRQ) 11-item checklist for cross-sectional studies(Pequeno, Cabral, Marchioni, Lima, & Lyra, 2020).

1. Source of information

The study should clearly state the data sources used (e.g., survey, registry, medical records).

2. Inclusion/Exclusion criteria stated or referenced

The study should describe or reference the inclusion and exclusion criteria for participants.

3. Time period specified

This item requires the study to provide a clear time frame during which the research was conducted.

4. Consecutive subjects if not population-based

This refers to whether the included subjects come from the same time period. Generally, if the study population is a whole population, continuity is guaranteed. In multicenter studies, it should be ensured that participants from all centers are recruited consecutively, since cross-sectional studies collect data over a continuous period.

5. Blinding of evaluators

This item assesses whether the researcher's subjective influence could obscure other aspects of the study participants. Evaluators should not make subjective assumptions but should base their assessment strictly on the actual data reported in the study. For example, the researcher should not judge a participant's expected score as higher than actual.

6. Quality assurance

This refers to quality control measures for outcome indicators, e.g., taking multiple measurements (such as blood pressure) and using the average value.

7. Reasons for exclusions from analysis

This item mainly concerns explanations for excluding certain populations. For example, if participants with psychiatric disorders were excluded, the study should explain why (e.g., inability to respond properly or affecting response quality).

8. Confounding assessed/controlled

Whether potential confounders are considered and controlled. For example, in a study on smoking and lung cancer, confounders such as age should be accounted for either in the design or by using statistical methods.

9. Missing data handled

The study should explain how missing data were managed.

10. Response rate & completeness of data collection

The study should report the response rate and completeness of data collection.

11. If follow-up, proportion with incomplete data or follow-up outcomes

If follow-up is mentioned in a cross-sectional study, the study should report the follow-up outcomes and rates; if not mentioned, it can be left unclear.

Supplementary Table 1. Healthy reference diet, with possible ranges, for an intake of 2500 kcal/day(Willett et al., 2019).

	Macronutrient intake (possible range), g/day	Caloric intake, kcal/day
**Whole grains***		
Rice, wheat, corn, and other[†]	232 (total grains 0–60% of energy)	811
**Tubers or starchy vegetables**		
Potatoes and cassava	50 (0–100)	39
**Vegetables**		
All vegetables	300 (200–600)	.
Dark green vegetables	100	23
Red and orange vegetables	100	30
Other vegetables	100	25
**Fruits**		
All fruit	200 (100–300)	126
**Dairy foods**		
Whole milk or derivative equivalents (eg, cheese)	250 (0–500)	153
**Protein sources**[‡]		
Beef and lamb	7 (0–14)	15
Pork	7 (0–14)	15
Chicken and other poultry	29 (0–58)	62
Eggs	13 (0–25)	19
Fish[§]	28 (0–100)	40
**Legumes**		
Dry beans, lentils, and peas*	50 (0–100)	172
Soy foods	25 (0–50)	112
Peanuts	25 (0–75)	142
Tree nuts	25	149
**Added fats**		
Palm oil	6·8 (0–6·8)	60
Unsaturated oils[¶]	40 (20–80)	354
Dairy fats (included in milk)	0	0
Lard or tallow[‖]	5 (0–5)	36

**Added sugars**		
All sweeteners	31 (0–31)	120

For an individual, an optimal energy intake to maintain a healthy weight will depend on body size and level of physical activity. Processing of foods such as partial hydrogenation of oils, refining of grains, and addition of salt and preservatives can substantially affect health but is not addressed in this table.

* Wheat, rice, dry beans, and lentils are dry, raw.

† Mix and amount of grains can vary to maintain isocaloric intake.

‡ Beef and lamb are exchangeable with pork and vice versa. Chicken and other poultry is exchangeable with eggs, fish, or plant protein sources. Legumes, peanuts, tree nuts, seeds, and soy are interchangeable.

§ Seafood consist of fish and shellfish (eg, mussels and shrimps) and originate from both capture and from farming. Although seafood is a highly diverse group that contains both animals and plants, the focus of this report is solely on animals.

¶ Unsaturated oils are 20% each of olive, soybean, rapeseed, sunflower, and peanut oil.

‖ Some lard or tallow are optional in instances when pigs or cattle are consumed.

Supplementary Table 2. Databases included in the Web of Science Core Collection.

Database Category	Database / Index Name	Notes
Journal Citation Indexes	Science Citation Index Expanded (SCIE)	Journals in natural sciences, engineering, and medicine
Journal Citation Indexes	Social Sciences Citation Index (SSCI)	Journals in the social sciences
Journal Citation Indexes	Arts & Humanities Citation Index (AHCI)	Journals in arts and humanities
Journal Citation Indexes	Emerging Sources Citation Index (ESCI)	Emerging journals under evaluation for flagship indexes
Conference Proceedings	Conference Proceedings Citation Index – Science (CPCI-S)	Conference papers in science & technology
Conference Proceedings	Conference Proceedings Citation Index – Social Sciences & Humanities (CPCI-SSH)	Conference papers in social sciences & humanities
Book Citation Indexes	Book Citation Index – Science (BKCI-S)	Scholarly books and book chapters (science)
Book Citation Indexes	Book Citation Index – Social Sciences & Humanities (BKCI-SSH)	Scholarly books and book chapters (SS&H)
Chemistry Indexes	Current Chemical Reactions (CCR Expanded)	Chemistry-focused index
Chemistry Indexes	Index Chemicus (IC)	Chemistry-focused index

From：

https://clarivate.com.cn/academia-government/scientific-and-academic-research/research-discovery-and-referencing/web-of-science/web-of-science-core-collection/

Supplementary Table 3. Literature search strategy and outcomes (coverage: from the inception to September 4, 2025)

Database	Search content	Number of retrieved items
PubMed	"EAT-Lancet"[Title/Abstract] OR "plant diet"[Title/Abstract] OR "planetary health diet"[Title/Abstract]	532
Web of Science	TI=("EAT-Lancet") OR TI=("plant diet") OR TI=("planetary health diet") OR AB=("EAT-Lancet") OR AB=("plant diet") OR AB=("planetary health diet")	850
Embase	'eat-lancet':ti,ab OR 'plant diet':ti,ab OR 'planetary health diet':ti,ab	613
Scopus	TITLE-ABS-KEY ("EAT-Lancet" OR "plant diet" OR "planetary health diet")	1,017
ProQuest Dissertations & Theses Global	TI,AB("EAT-Lancet") OR TI,AB("plant diet") OR TI,AB("planetary health diet")	48
Total	/	3,060

These databases were selected to ensure comprehensive coverage of the biomedical and multidisciplinary literature relevant to diet and neuropsychiatric outcomes. PubMed provides core coverage of biomedical research; Embase offers broader European and pharmacological indexing and includes additional journals and conference records not fully captured in PubMed; Web of Science (Core Collection) and Scopus provide extensive multidisciplinary coverage and citation tracking to help identify relevant studies across disciplines and to facilitate forward/backward citation searching. ProQuest Dissertations & Theses Global was searched to capture grey literature (e.g., theses/dissertations), thereby reducing the risk of publication bias and improving the completeness of the evidence base.

Supplementary Table 4. Summary of EAT-Lancet/Planetary Health Diet adherence scoring methods.

Scoring method	Number of components	Components	Scoring approach
Knuppel score 0–14	14	Whole grains; potatoes; vegetables; fruits; dairy; beef lamb pork; poultry; eggs; fish; dry beans lentils peas; soy foods; peanuts and tree nuts; added fats as unsaturated to saturated fat ratio; added sugars	Binary scoring. Each component scores 1 if the intake meets the cut-off, otherwise 0. Total is the sum, 0–14. Cut-offs in g per day: whole grains up to 464; potatoes up to 100; vegetables at least 200; fruits at least 100; dairy up to 500; beef lamb pork up to 28; poultry up to 58; eggs up to 25; fish up to 100; dry beans lentils peas up to 100; soy foods up to 50; peanuts and tree nuts at least 25; added sugars up to 31. Added fats score 1 if the unsaturated to saturated fat ratio is at least 0.8.
Colizzi score 0–140	14	Whole grains; vegetables; fruits; potatoes and cassava; dairy; dry beans lentils peas; soy foods; beef lamb pork; poultry; eggs; fish; nuts; added sugars; added fats as unsaturated to saturated fat ratio	Proportional scoring. Each component is scored 0–10, summed to 0–140. Components are scored according to the EAT-Lancet target, using linear increases for adequacy components, linear decreases for moderation components, peak scoring within a target interval for optimum components, and a ratio-based score for added fats using the unsaturated to saturated fat ratio.
Stubbendorff score 0–42	14	Vegetables; fruits; unsaturated oils; legumes; nuts; whole grains; fish; beef and lamb; pork; poultry; eggs; dairy; potatoes; added sugar	Ordinal scoring with fixed intake bands. Each component scores 0–3 and totals 0–42. All cut-offs in g per day. Emphasized components score higher with higher intake: vegetables >300 scores 3, 200–300 scores 2, 100–200 scores 1, <100 scores 0; fruits >200 scores 3, 100–200 scores 2, 50–100 scores 1, <50 scores 0; unsaturated oils >40 scores 3, 20–40 scores 2, 10–20 scores 1, <10 scores 0; legumes >75 scores 3, 37.5–75 scores 2, 18.75–37.5 scores 1, <18.75 scores 0; nuts >50 scores 3, 25–50 scores 2, 12.5–25 scores 1, <12.5 scores 0; whole grains >232 scores 3, 116–232 scores 2, 58–116 scores 1, <58 scores 0; fish >28 scores 3, 14–28 scores 2, 7–14 scores 1, <7 scores 0. Limited components score higher with lower intake: beef and lamb <7 scores 3, 7–14 scores 2, 14–28 scores 1, >28 scores 0; pork <7 scores 3, 7–14 scores 2, 14–28 scores 1, >28 scores 0; poultry <29 scores 3, 29–58 scores 2, 58–116 scores 1, >116 scores 0; eggs <13 scores 3, 13–25 scores 2, 25–50 scores 1, >50 scores 0; dairy <250 scores 3, 250–500 scores 2, 500–1000 scores 1, >1000 scores 0; potatoes <50 scores 3, 50–100 scores 2, 100–200 scores 1, >200 scores 0; added sugar <31 scores 3, 31–62 scores 2, 62–124 scores 1, >124 scores 0.

Other ELD, PHDI, PHDI-US variants	14–16 varies by index	ELD also called Kesse-Guyot EAT-Lancet index: 14 food groups aligned to EAT-Lancet cut-offs; PHDI and PHDI-US: 16 components spanning plant foods, animal-source foods, fats, and added sugars; includes adequacy, optimum, ratio, and moderation domains	ELD: continuous deviation score based on cut-offs, computed after scaling intakes to a 2500 kcal diet; deviations are signed by component direction and summed across 14 components; the total can be negative. PHDI: 16 components with a total score 0–150; each component scores up to 10 or 5 points using proportional scoring based on energy contribution and EAT-Lancet reference targets; components are grouped as adequacy, optimum, ratio, and moderation. PHDI-US: adaptation of PHDI for US dietary data; 16 components with total score 0–150.

Diet score operationalizations may vary across cohorts/studies (e.g., food group definitions, cut-points, energy adjustment, and component scaling). The descriptions above summarize the commonly reported structure of each scoring approach and should be interpreted as guidance; please refer to each original study’s methods/supplementary materials for exact implementation details.

Supplementary Table 5. Summary of outcome diagnostic criteria and assessment methods.

Outcome			Assessment criteria or tools*
Disease or disorder	Depressive disorder, Anxiety disorder, Schizophrenia, Bipolar, Stroke, Dementia.		DSM-III, DSM-III-R, DSM-IV, DSM-IV-TR, DSM-5; ICD-9, ICD-10, ICD-11
Symptoms	Depressive symptoms		PHQ-9; HAMD; MADRS; BDI; CES-D; SDS; HADS; DASS-21
	Anxiety symptoms		GAD-7; HAMA; BAI; STAI; SAS; HADS; DASS-21
	Cognitive decline	Global cognition	MMSE; MoCA; SM-MMSE; 3MS; ACE-III; RBANS
		Composite cognition	Neuropsychological test battery
		Episodic memory	RAVLT; 15WT; CERAD word list; word list recall and recognition
		Executive function	TMT-A; TMT-B; Stroop; verbal fluency tasks
		Processing speed	SDMT; Coding task; TMT-A
		Working memory	Digit Span; Digit Span Backward
		Language	Semantic fluency; phonemic fluency; naming tasks
		Visuospatial ability	Block design; clock drawing tasks

*Cut-off values for symptom scales were defined in the original studies. DSM-III = Diagnostic and Statistical Manual of Mental Disorders, Third Edition; DSM-III-R = Diagnostic and Statistical Manual of Mental Disorders, Third Edition, Revised; DSM-IV = Diagnostic and Statistical Manual of Mental Disorders, Fourth Edition; DSM-IV-TR = Diagnostic and Statistical Manual of Mental Disorders, Fourth Edition, Text Revision; DSM-5 = Diagnostic and Statistical Manual of Mental Disorders, Fifth Edition; ICD-9 = International Classification of Diseases, 9th Revision; ICD-10 = International Classification of Diseases, 10th Revision; ICD-11 = International Classification of Diseases, 11th Revision; PHQ-9 = Patient Health Questionnaire-9; HAMD = Hamilton Depression Rating Scale; MADRS = Montgomery–Åsberg Depression Rating Scale; BDI = Beck Depression Inventory; CES-D = Center for Epidemiologic Studies Depression Scale; SDS = Zung Self-Rating Depression Scale; HADS = Hospital Anxiety and Depression Scale; DASS-21 = Depression, Anxiety and Stress Scale, 21-item version; GAD-7 = Generalized Anxiety Disorder-7; HAMA = Hamilton Anxiety Rating Scale; BAI = Beck Anxiety Inventory; STAI = State–Trait Anxiety Inventory; SAS = Zung Self-Rating Anxiety Scale; MMSE = Mini-Mental State Examination; MoCA = Montreal Cognitive Assessment; SM-MMSE = Singapore-modified Mini-Mental State Examination; 3MS = Modified Mini-Mental State Examination; ACE-III = Addenbrooke's Cognitive Examination III; RBANS = Repeatable Battery for the Assessment of Neuropsychological Status; RAVLT = Rey Auditory Verbal Learning Test; 15WT = 15 Word Test; CERAD = Consortium to Establish a Registry for Alzheimer's Disease; TMT = Trail Making Test; SDMT = Symbol Digit Modalities Test.

Supplementary Table 6. Summary of study designs and key characteristics of the included studies.

Item	Summary
Total included studies	28 studies
Study design	22 cohort studies; 6 cross-sectional studies
Outcomes (number of included studies)	Depression (6 studies); Anxiety (3 studies); Stroke (12 studies); Cognition (5 studies); Dementia (2 studies); Cognitive impairment (2 studies)
Outcome data type	Cognitive function: continuous measure; all other outcomes: binary
Geographic region	Europe (15 studies); Americas (7 studies); Asia (6 studies)
Repeated cohorts	UK Biobank (stroke, 4 studies); NHANES (depression, 3 studies)
Further details	Detailed study characteristics are presented in Table 1

National Health and Nutrition Examination Survey (NHANES).

Supplementary Table 7. Assessment of publication bias using Egger's test.

Outcome	Model	Number of studies	Egger intercept (beta1)	SE	z	*P* value
Anxiety	Fixed effects	3	-0.12	1.106	-0.11	0.9116
Depression	Fixed effects	6	-1.37	0.969	-1.42	0.157
Stroke	Random effects	12	0.93	1.675	0.56	0.5778
Dementia	Fixed effects	2	-3.56	6.441	-0.55	0.5804
Cognition	Random effects	4	2.85	1.018	2.8	0.0051

Supplementary Table 8. Quality assessment overview of the Cohort studies.

Study	Selection				Comparability		Outcome or exposure assessment			NOS score
	Representative of the exposed cohort	Selection of the non exposed cohort	Ascertainment of exposure	Demonstration that outcomes of interest was not present at start of study	Comparability of cohorts on the basis of the design or analysis	Comparability of cohorts on the basis of the measurement	Assessment of outcomes	Was follow-up long enough for outcomes to occur	Adequacy of follow up of cohorts	
Knuppel et al., 2019*	★	NA	☆	NA	★	★	★	NA	NA	NA
Ibsen et al., 2022	★	★	☆	★	★	★	★	★	★	8.5
Berthy et al., 2022	★	★	☆	★	★	★	★	★	★	8.5
Colizzi et al., 2023	★	★	☆	★	★	★	★	★	★	8.5
Karavasiloglou et al., 2023	★	★	☆	★	★	★	★	★	★	8.5
Lu et al., 2024	★	★	☆	★	★	★	★	★	★	8.5
Sawicki et al., 2024	★	★	☆	★	★	★	★	★	★	8.5
Ye et al., 2024	★	★	☆	★	★	★	★	★	★	8.5
Zhang et al., 2024	★	★	☆		★	★	★	★		6.5
van Soest et al., 2024	☆	★	☆		★	★	★			5
Gomes	★	★	☆	★	★	★	★	★		7.5

Gonçalves et al., 2024										
Stubbendorff et al., 2024	★	★	☆	★	★	★	★	★	★	8.5
Colizzi et al., 2024*	★	NA	☆	NA	★	★	★	NA	NA	NA
Karavasiloglou et al., 2025	★	★	☆	★	★	★	★	★	★	8.5
Sotos-Prieto et al., 2025	★	★	☆	★	★	★	★	★	★	8.5
Zhao et al., 2025	★	★	☆	★	★	★	★	★	★	8.5
Samuelsson et al., 2025	★	★	☆	★	★	★	★	★	★	8.5
Wu et al., 2025	★	★	☆	★	★	★	★	★		7.5
Li et al., 2025	★	★	☆	★	★	★	★	★		7.5
Wijnhoven et al., 2025	★	★	☆	★	★	★	★	★	★	8.5
Tang et al., 2025	★	★	☆	★	★	★	★	★		7.5
Yang et al., 2025*	★	NA	☆	NA	★	★	★	NA	NA	NA

★ = Item fulfilled, scored as 1 point; ☆ = Partially fulfilled, scored as 0.5 point; (Blank) = Item not clearly reported in the full text and therefore scored as 0 point; NA = Not assessable because only the abstract was available; * = Only the abstract.

Supplementary Table 9. Quality assessment overview of the Cross-sectional studies.

Study	Source of information	Inclusion /Exclusion criteria stated or referenced	Time period specified	Consecutive subjects if not population-based	Blinding of evaluators	Quality assurance	Reasons for exclusions from analysis	Confounding assessed /controlled	Missing data handled	Response rate & completeness of data collection	If follow-up, proportion with incomplete data or follow-up outcomes	AHRQ 11-item score
Kamrani et al., 2024	★	★	★	★	★	★	★	★	★		†	9
Jiang et al., 2025	★	★	★	★	★	★	★	★	★		†	9
Lan et al., 2025	★	★	★	★	★	★	★	★	★		†	9
Tabatabaei et al., 2025	★	★	★	★	★	★	★	★	★		†	9
Tan et al., 2025	★	★		★	★	★	★	★	★		†	9
Samuelsson et al., 2025	★	★	★	★	★	★	★	★	★	★	†	10

★ = Item fulfilled, scored as 1 point; (Blank) = Item not clearly reported and therefore scored as 0 point; † = Not applicable because all cross-sectional studies included in this

review did not involve follow-up, and this item was excluded from scoring.

Supplementary Table 10. Certainty of evidence for the associations between adherence to the EAT-Lancet diet and neuropsychiatric disorders with GRADE.

	Anxiety	Depression	Stroke	Dementia	Cognition
Total number of studies	3	6	12	2	5
Studies included in meta-analyses	3	6	12	2	4
Study design	Cross-sectional (n=2), Longitudinal (n=1)	Cross-sectional (n=5), Longitudinal (n=1)	Longitudinal	Longitudinal	Longitudinal
Number of participants	187,019	268,671	1,614,851	216,791	17,111
Effect Estimate (95% CI)	0.82 (0.76, 0.89)	0.76 (0.71, 0.81)	0.84 (0.76, 0.92)	0.96 (0.93, 1.00)	0.02 (-0.01, 0.06)
Limitations					
-Risk of bias	Not serious	Not serious	Not serious	Not serious	Not serious
-Inconsistency	Not serious	Not serious	Not serious	Not serious	Not serious
-Indirectness	Not serious	Not serious	Not serious	Not serious	Not serious
-Imprecision	Not serious	Not serious	Not serious	Not serious	Not serious
-Publication bias	Not suspected	Not suspected	Not suspected	Not suspected	Suspected
Strengths					
-Large Effect	No	No	No	No	No
-Dose-Response Gradient	No	No	No	No	No
-Plausible Confounding That Would Reduce the Effect	No	No	No	No	No
Potential upgrading or downgrading factors	No change	No change	No change	No change	Downgrade
Overall certainty	Very Low	Very Low	Low	Low	Very Low

A. Ischemic stroke

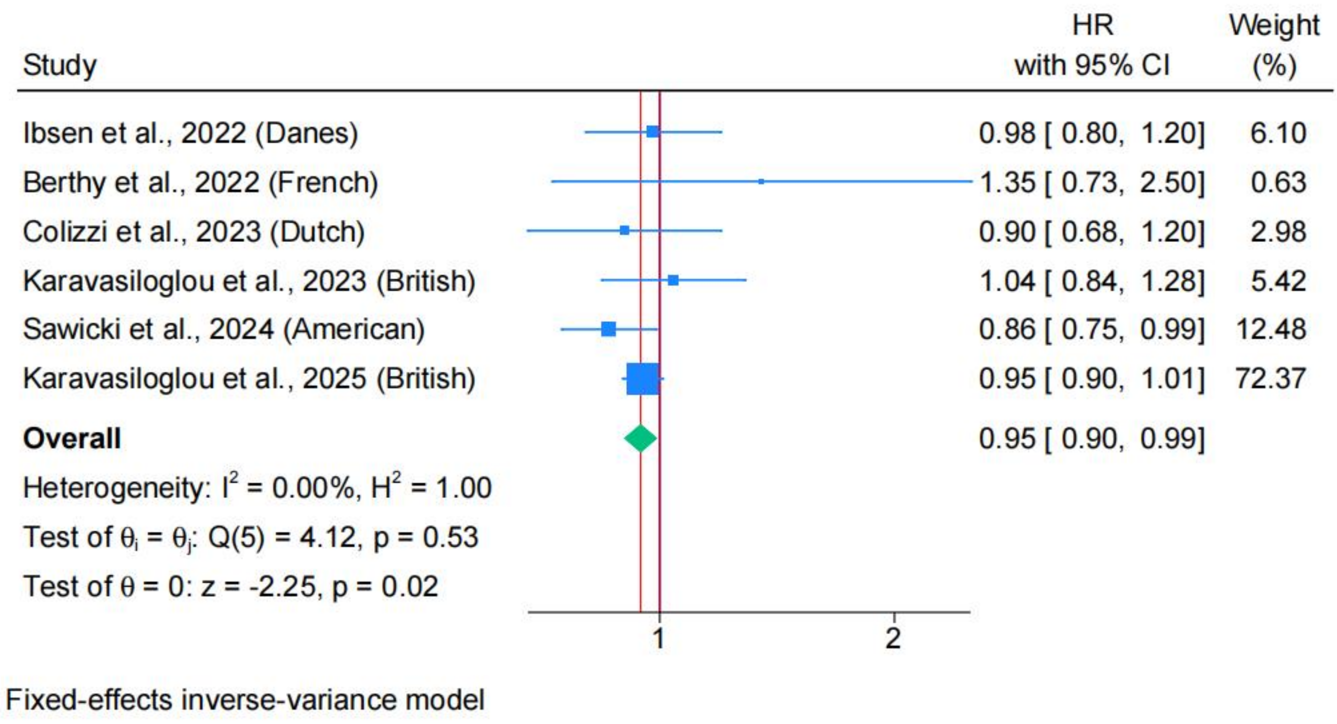

B. Hemorrhagic stroke

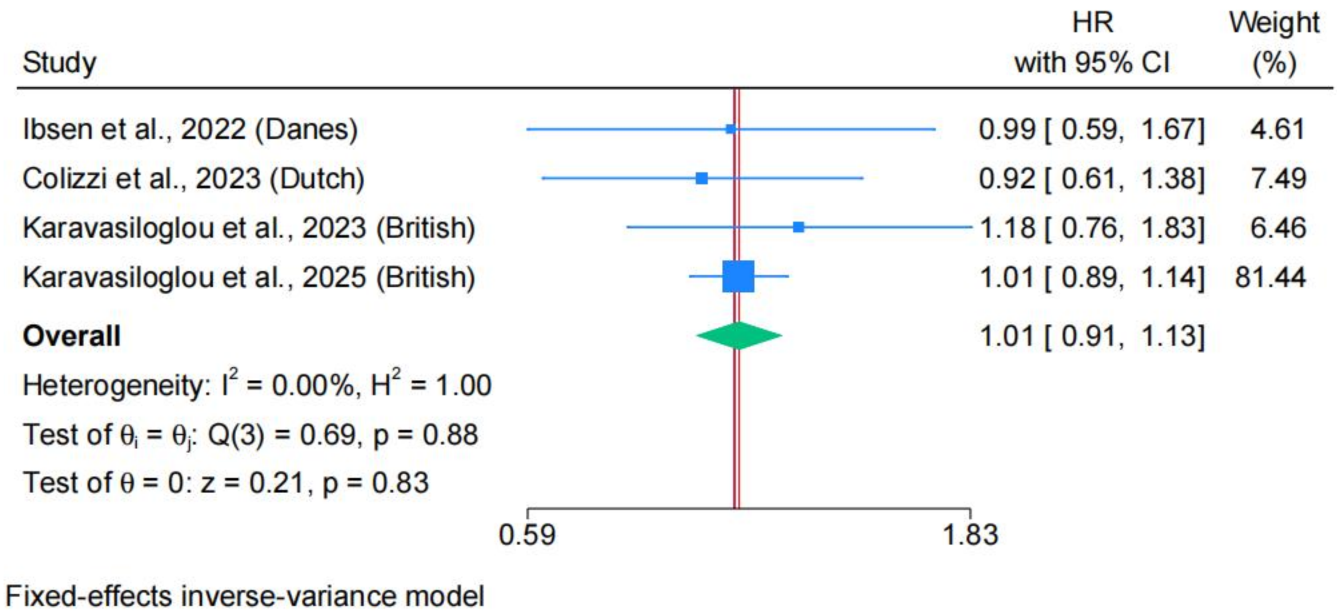

C. Depression (cross-sectional studies)

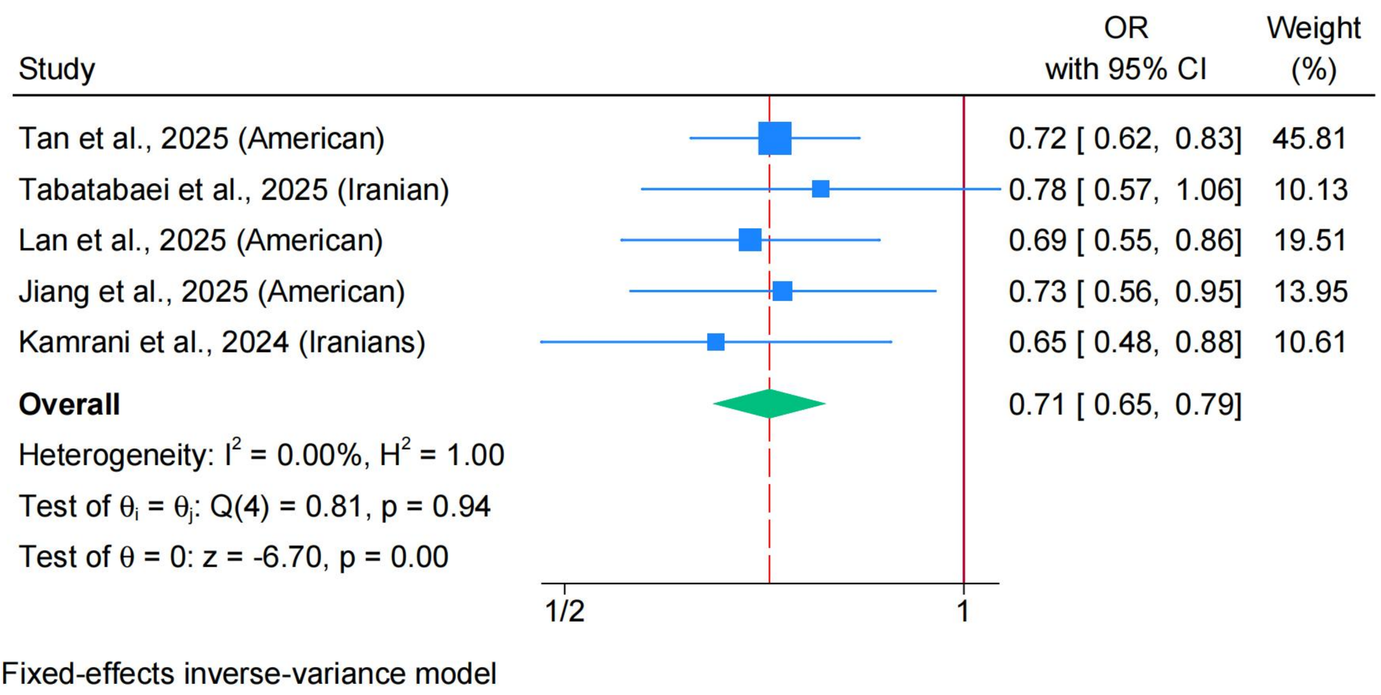

D. Depression (cohort study)

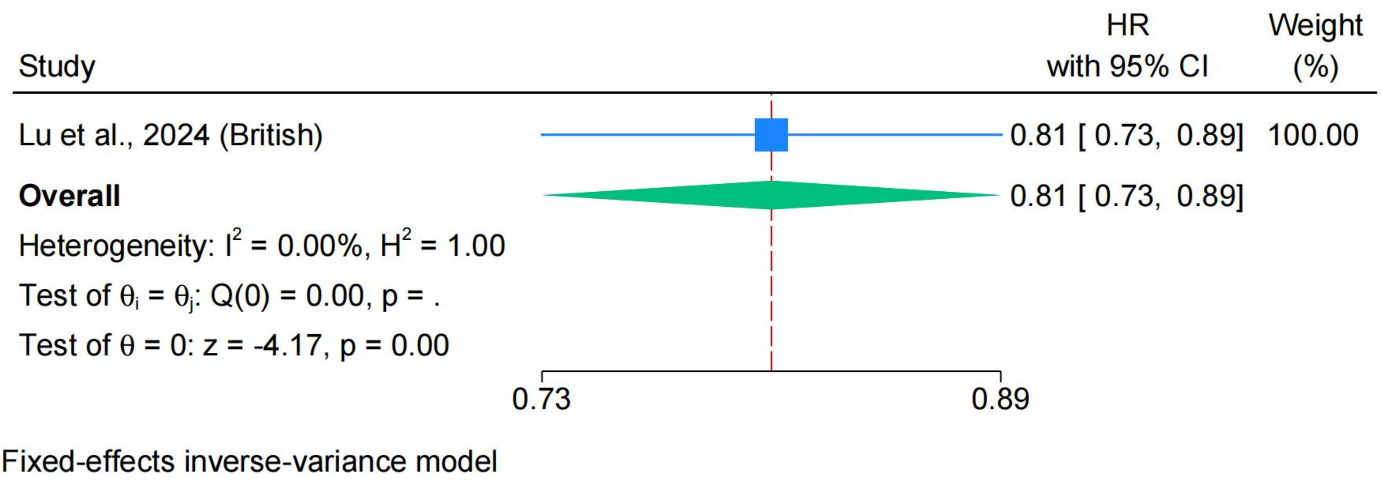

E. Anxiety (cross-sectional studies)

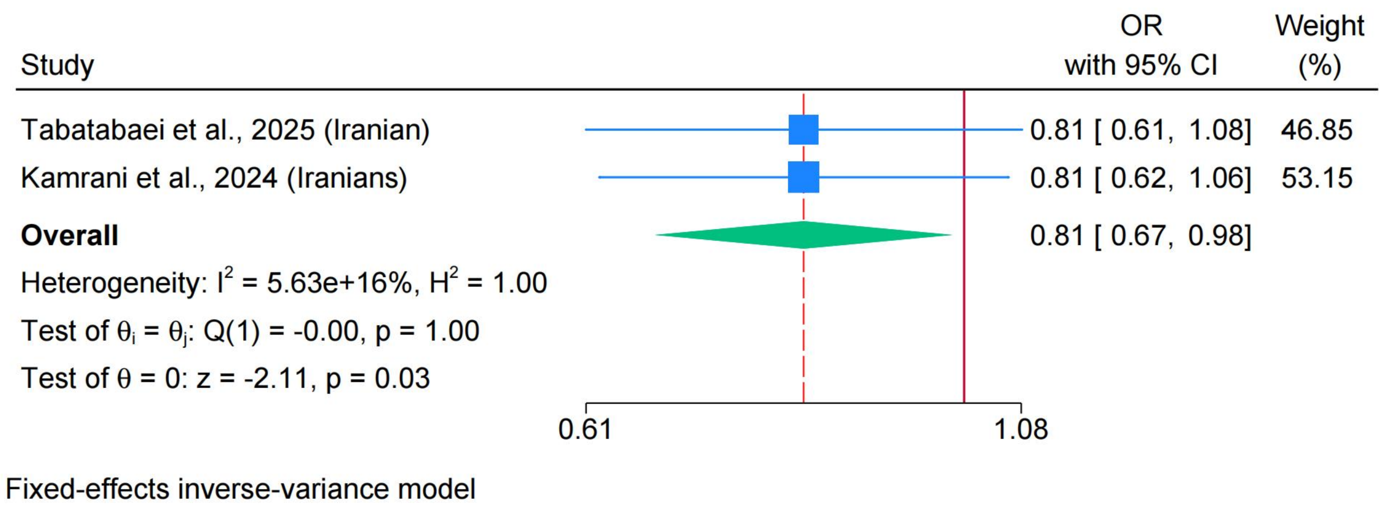

F. Anxiety (cohort study)

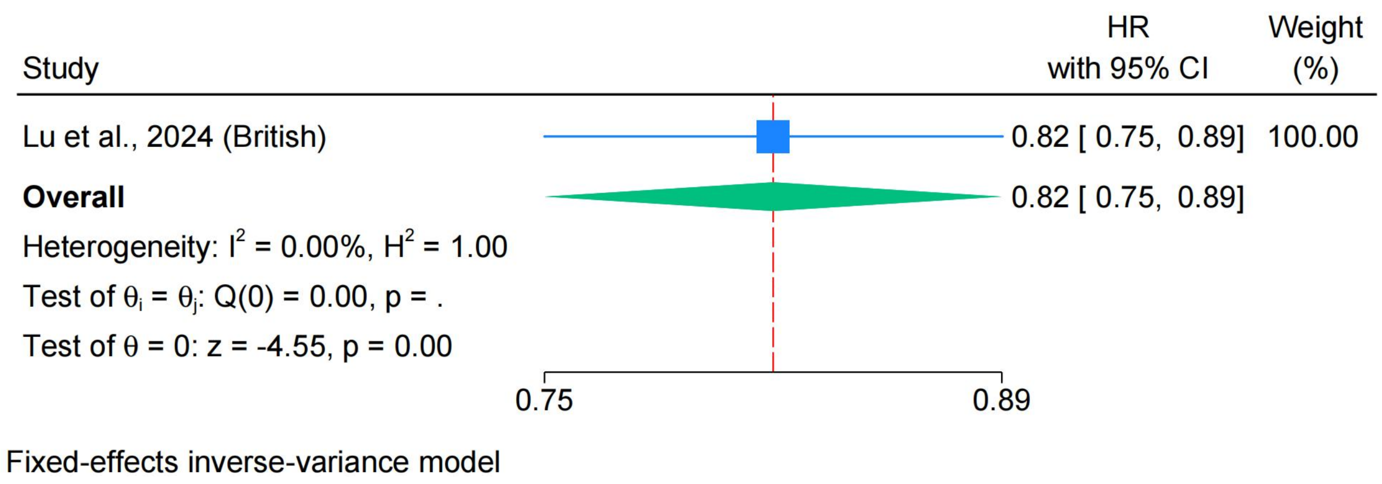

G. Cognition (change model)

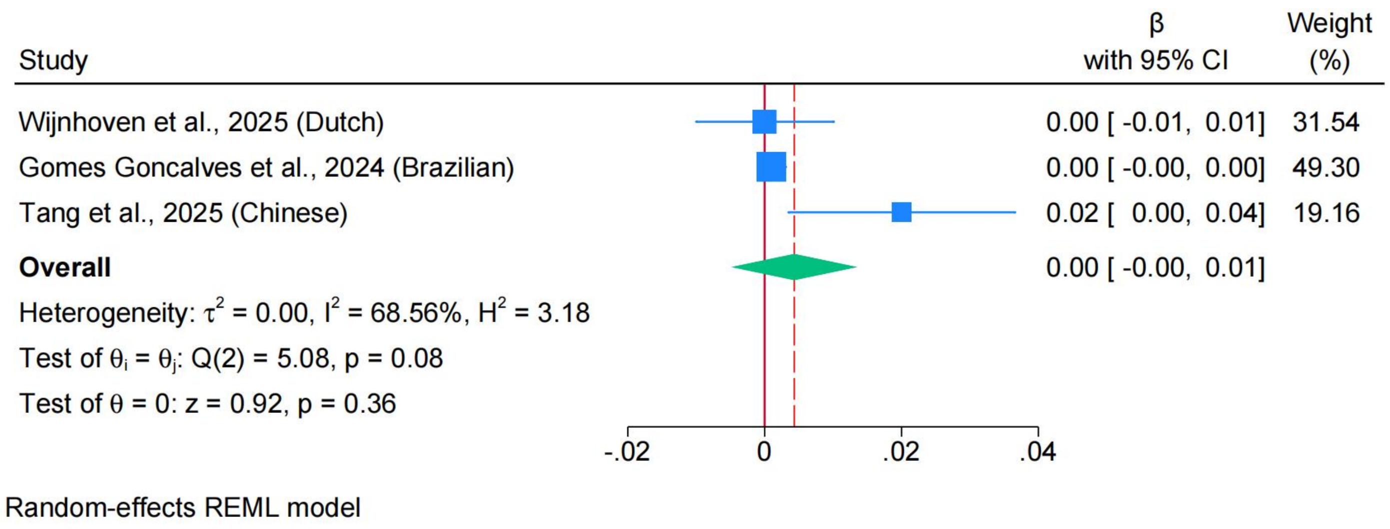

H. Cognition (level model)

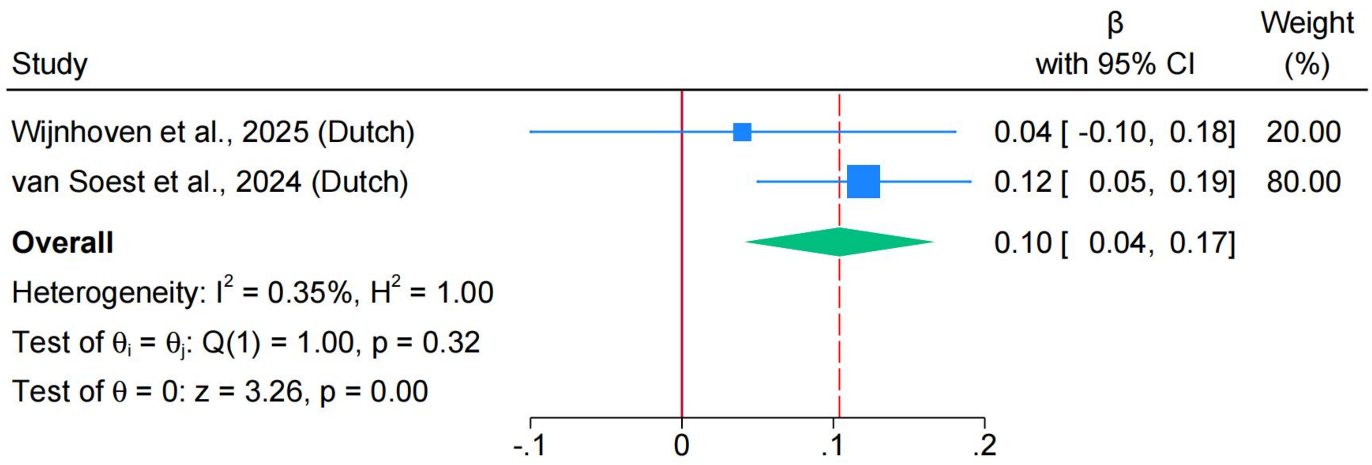

Fixed-effects inverse-variance model

I. Memory

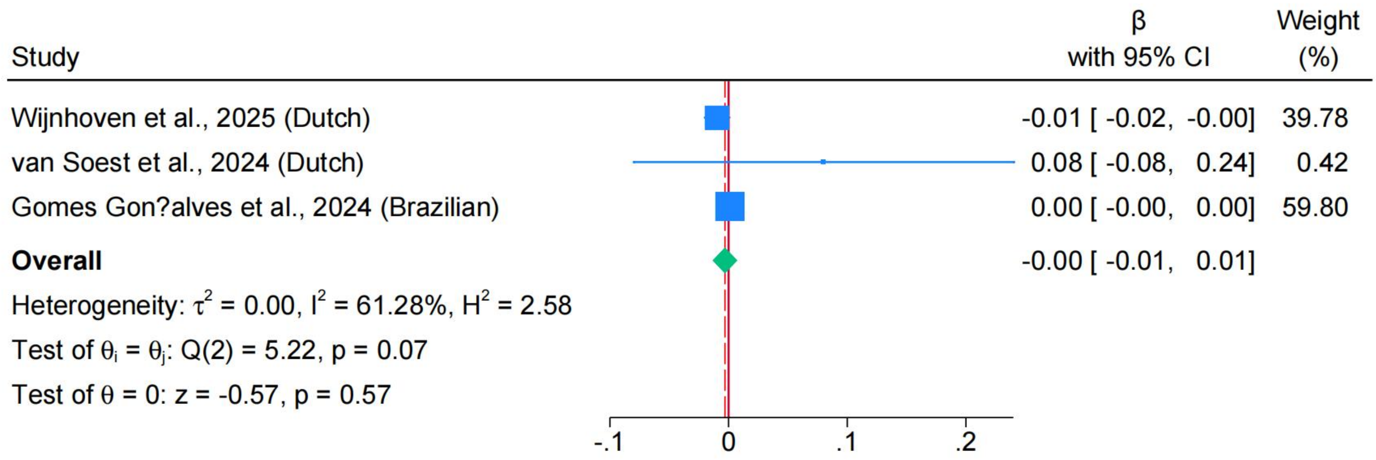

Random-effects REML model

J. Attention

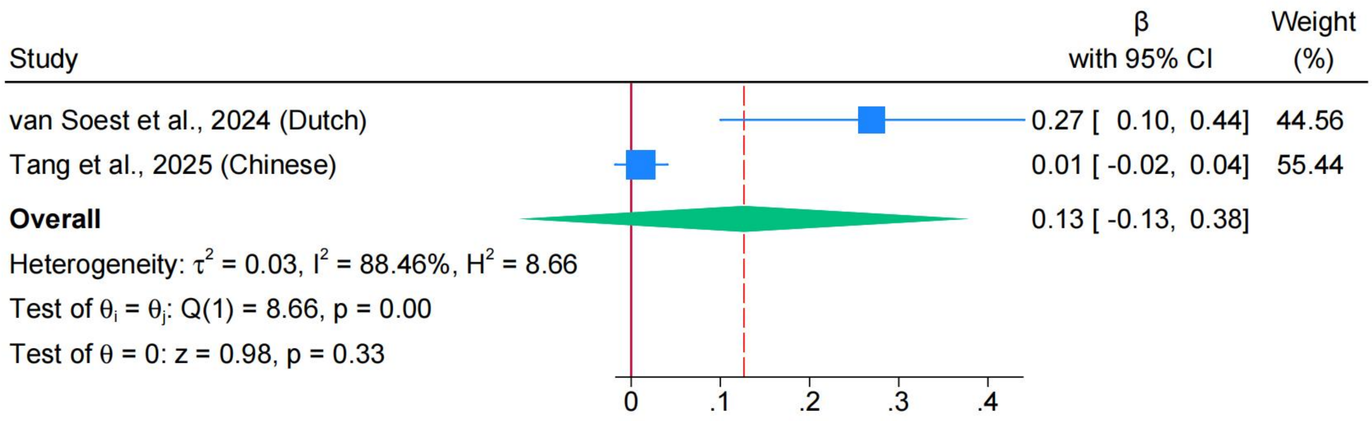

Random-effects REML model

K. Executive function

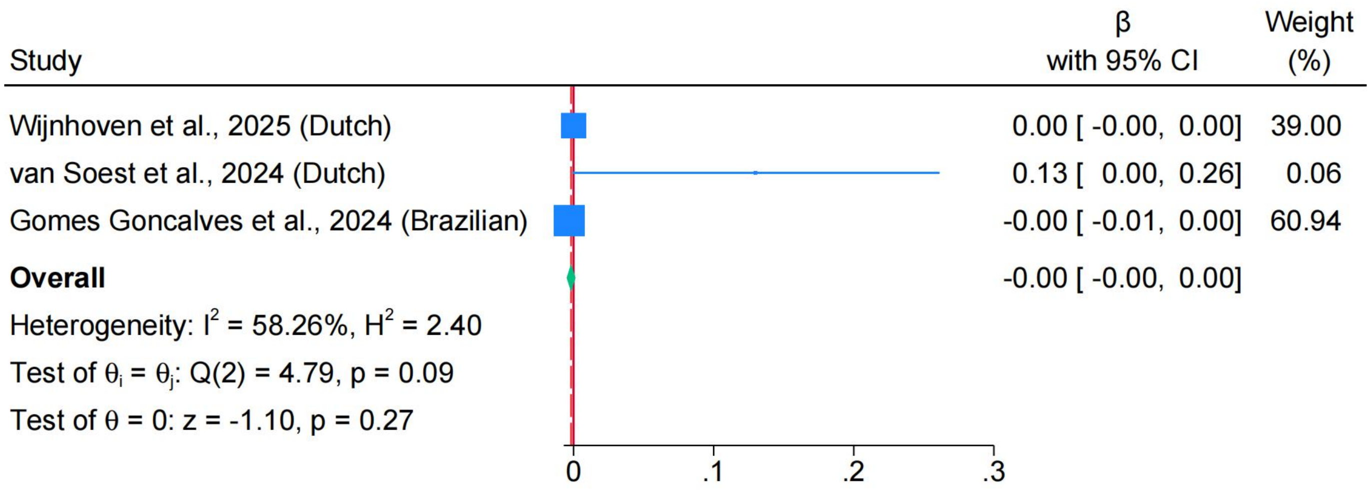

Fixed-effects inverse-variance model

L. Information processing speed

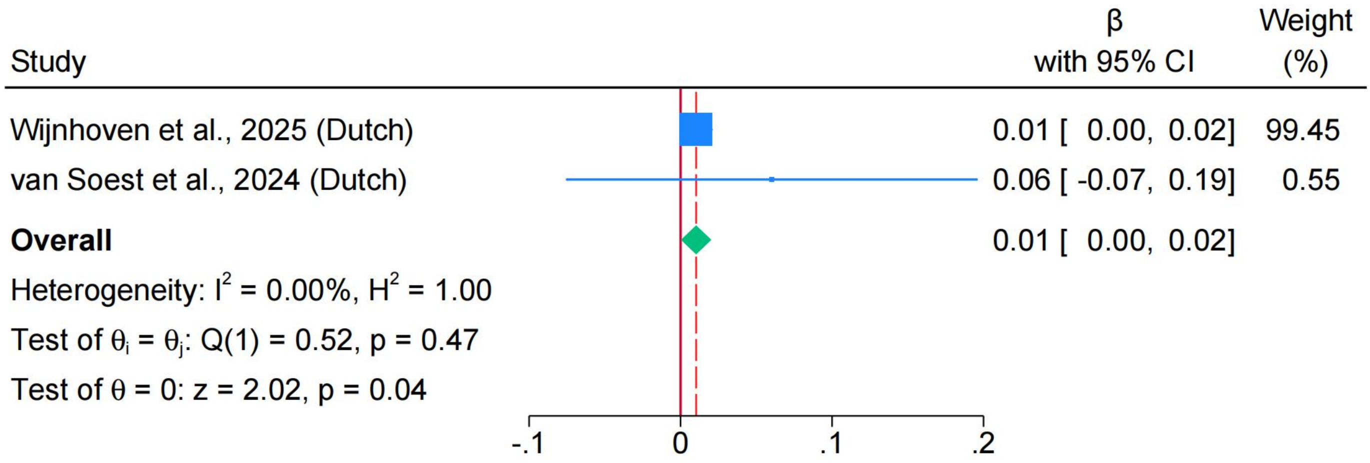

Fixed-effects inverse-variance model

M. Stroke (Knuppel score)

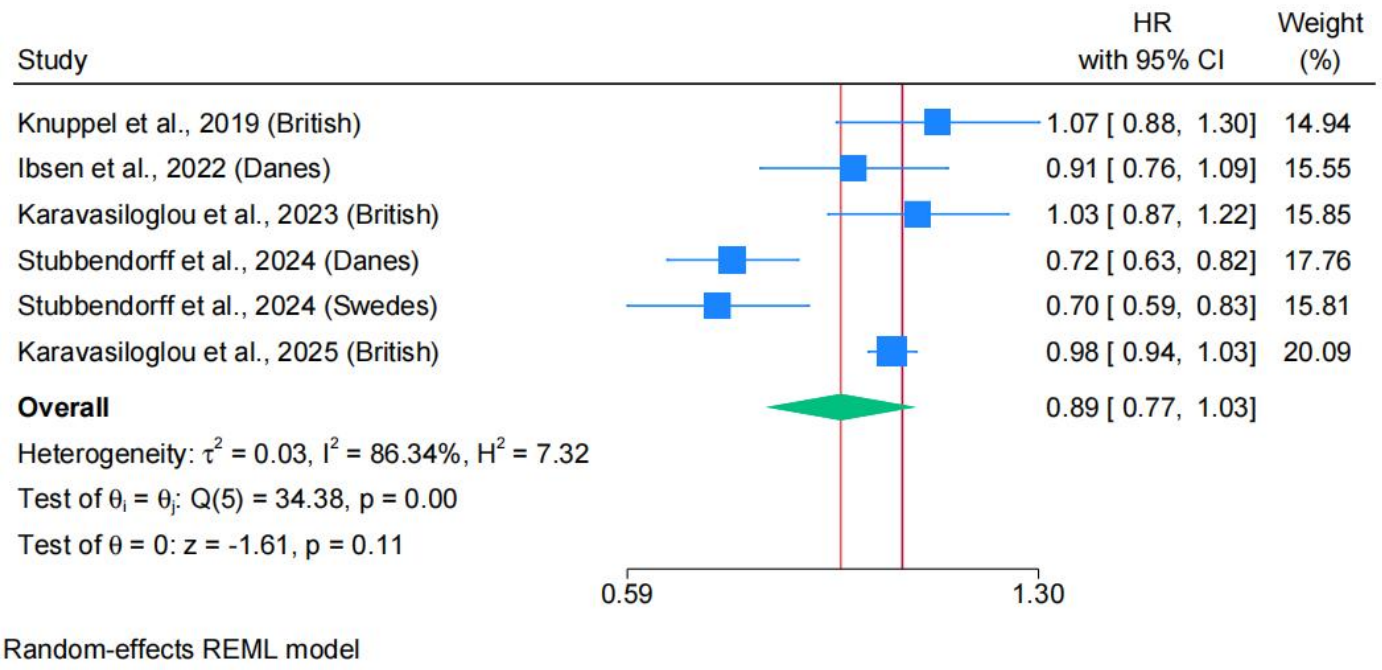

N.Stroke (other scores)

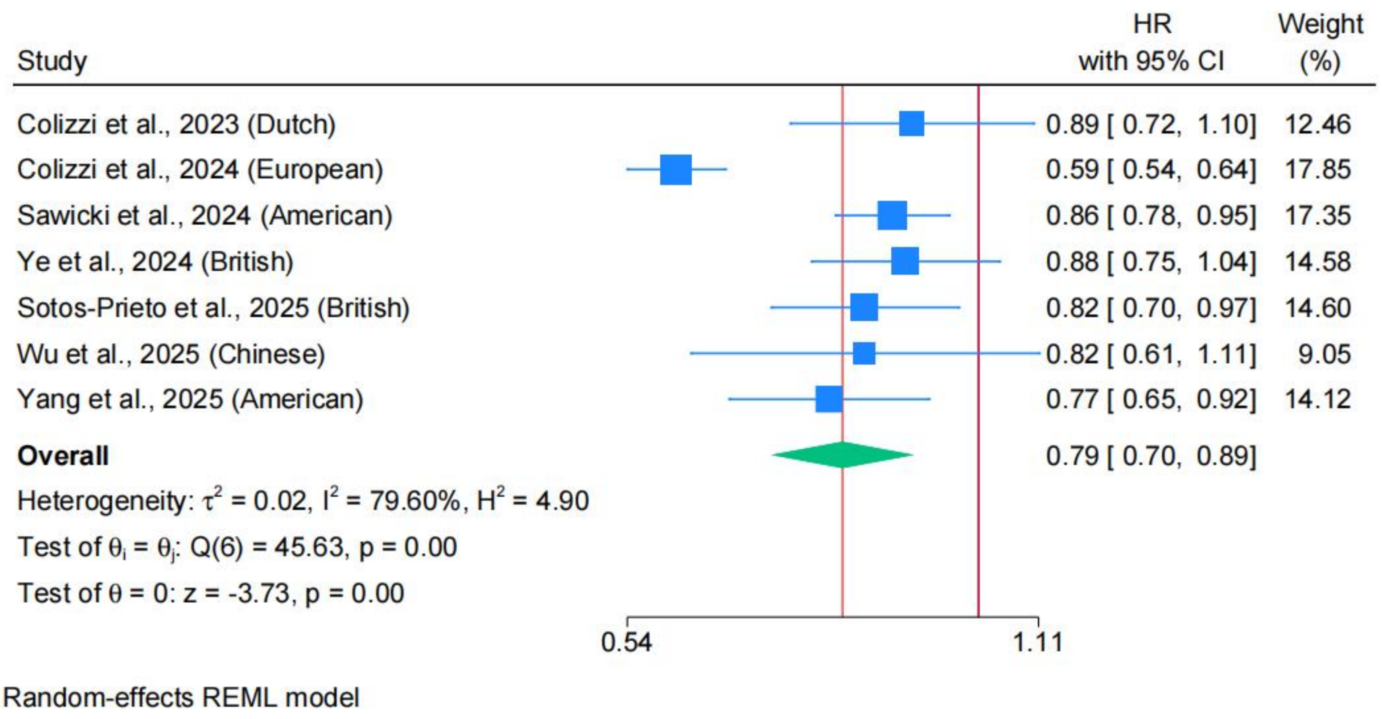

Supplementary Figure 1. The forest plots of meta-analysis for subgroup analyses. **(A)** Forest plot of ischemic stroke across included studies. **(B)** Forest plot of hemorrhagic stroke across included studies. **(C)** Forest plot of depression in cross-sectional studies. **(D)** Forest plot of depression in cohort studies. **(E)** Forest plot of anxiety in cross-sectional studies. **(F)** Forest plot of anxiety in cohort studies.**(G)** Forest plot of Change Model in cognitive scores. **(H)** Forest plot of Level Model in cognitive scores. **(I)** Forest plot of memory, a part of cognitive scores. **(J)** Forest plot of

attention, a part of cognitive scores. **(K)** Forest plot of executive function, a part of cognitive scores. **(L)** Forest plot of information processing speed, a part of cognitive scores. **(M)** Forest plot of total stroke based on the Knuppel score. **(N)** Forest plot of total stroke based on other scores, including Colizzi (0–140), PHDI (0–130, 0–150, 0–140), etc..

A

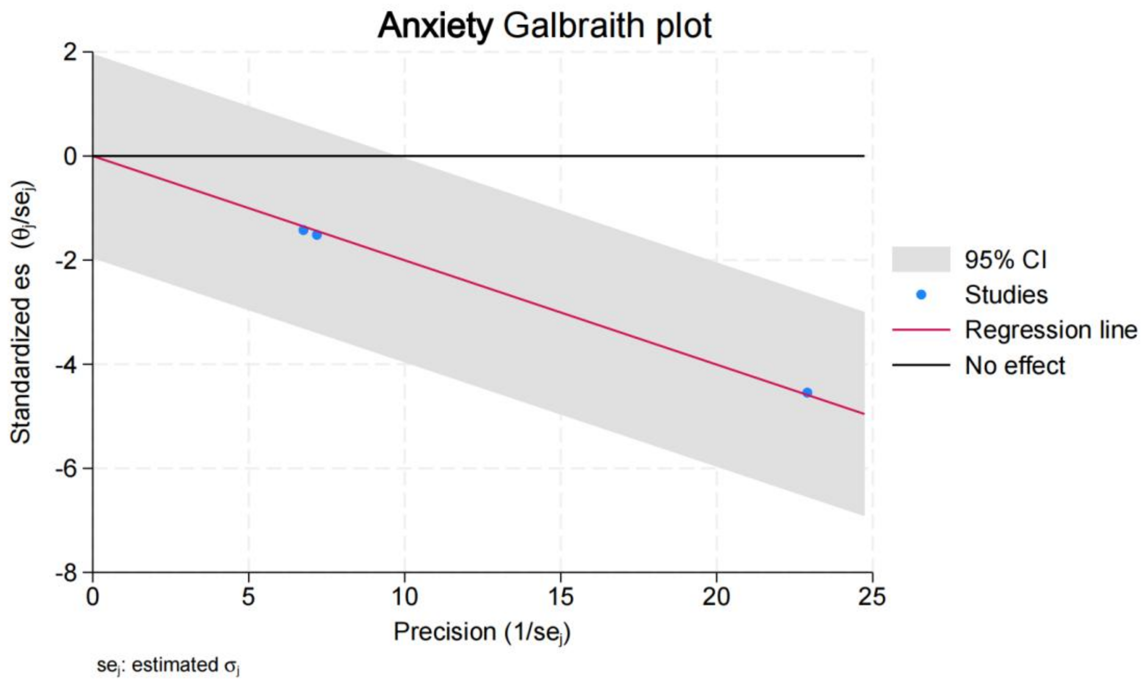
Anxiety Galbraith plot
2
0
-2
-4
-6
-8
Standardized es (θ_j/se_j)
0
5
10
15
20
25
Precision ($1/se_j$)
se_j: estimated σ_j
95% CI
Studies
Regression line
No effect

B

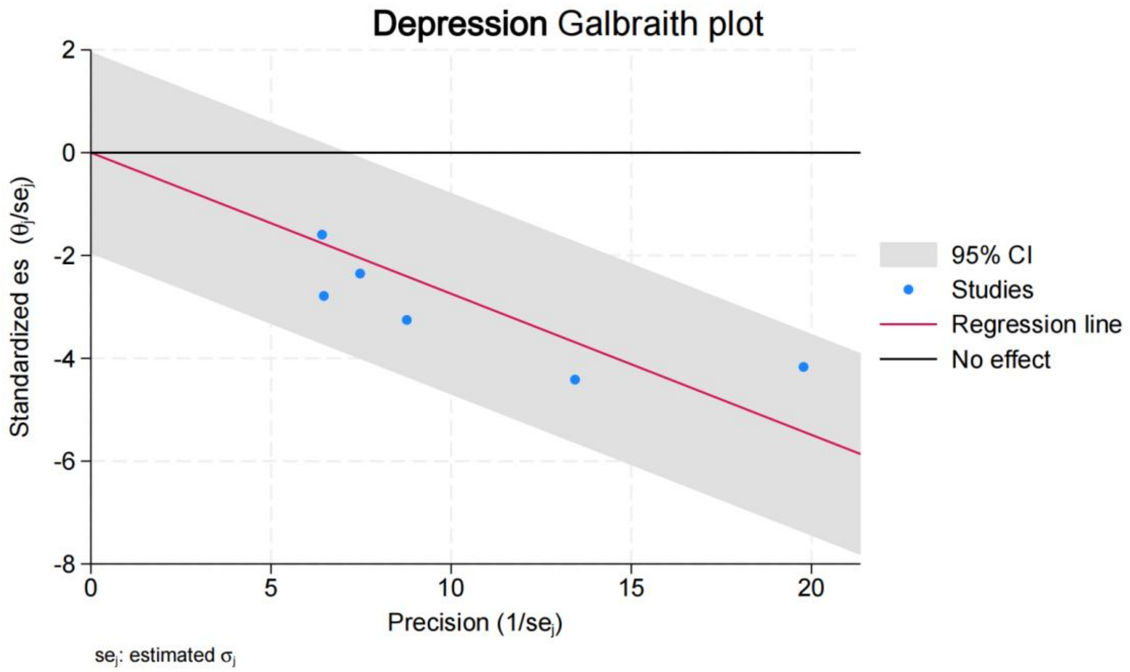
Depression Galbraith plot
2
0
-2
-4
-6
-8
Standardized es (θ_j/se_j)
0
5
10
15
20
Precision ($1/se_j$)
se_j: estimated σ_j
95% CI
Studies
Regression line
No effect

C

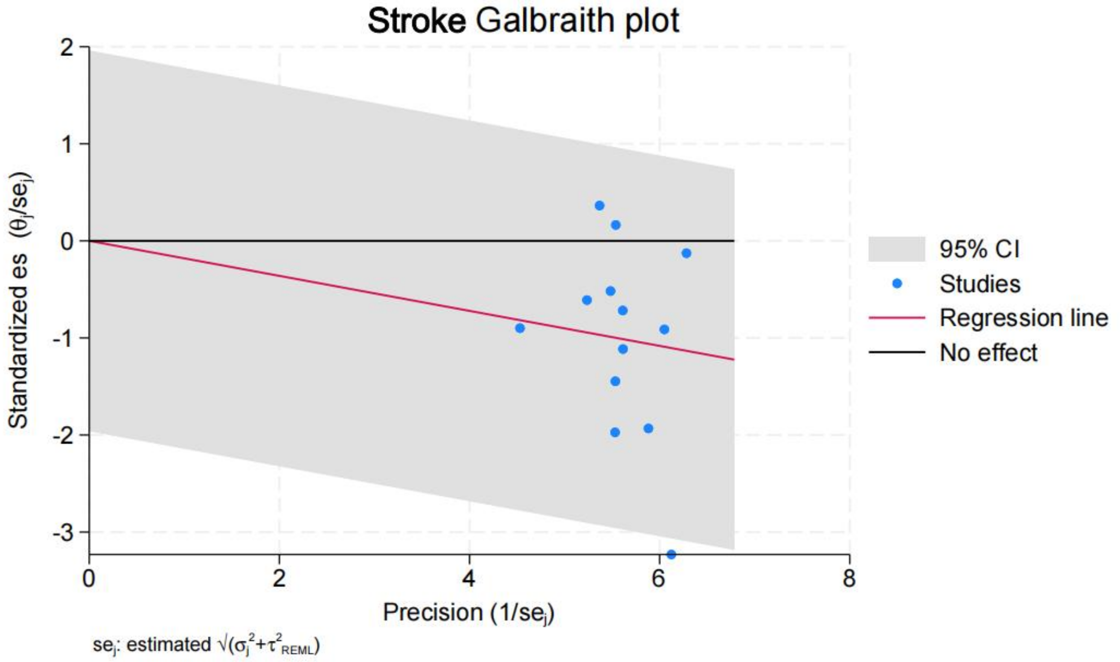

Stroke Galbraith plot
Standardized es (θj/sej)
Precision (1/sej)
95% CI
Studies
Regression line
No effect
sej: estimated √(σj²+τ²REML)

D

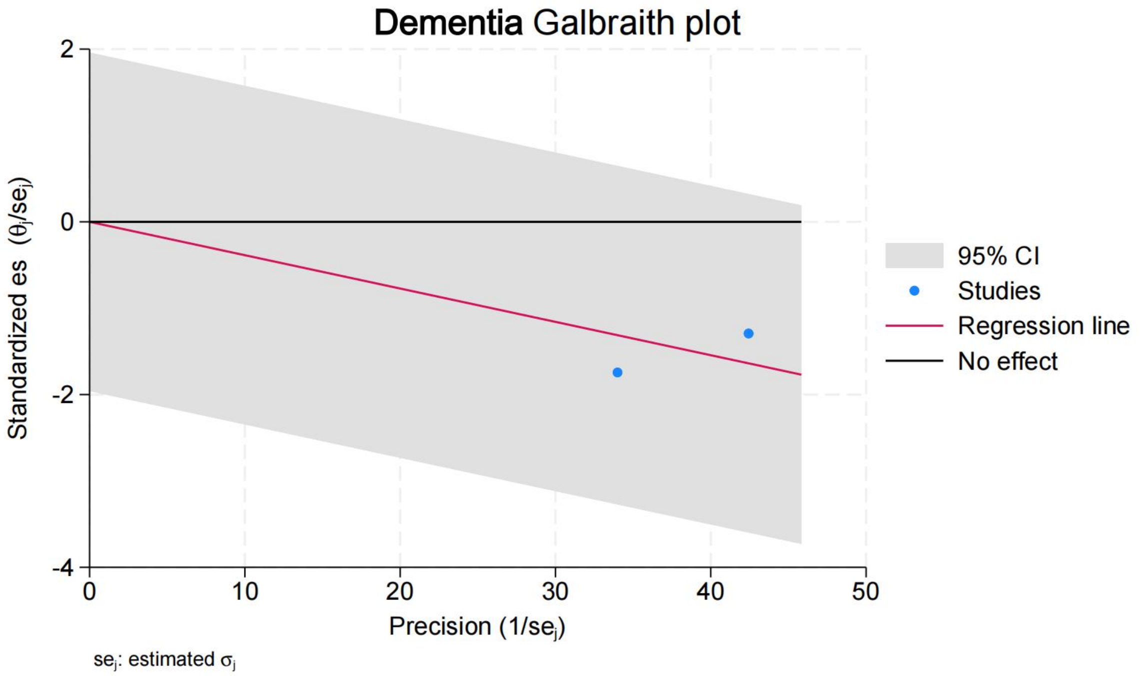

Dementia Galbraith plot
Standardized es (θj/sej)
Precision (1/sej)
95% CI
Studies
Regression line
No effect
sej: estimated σj

E

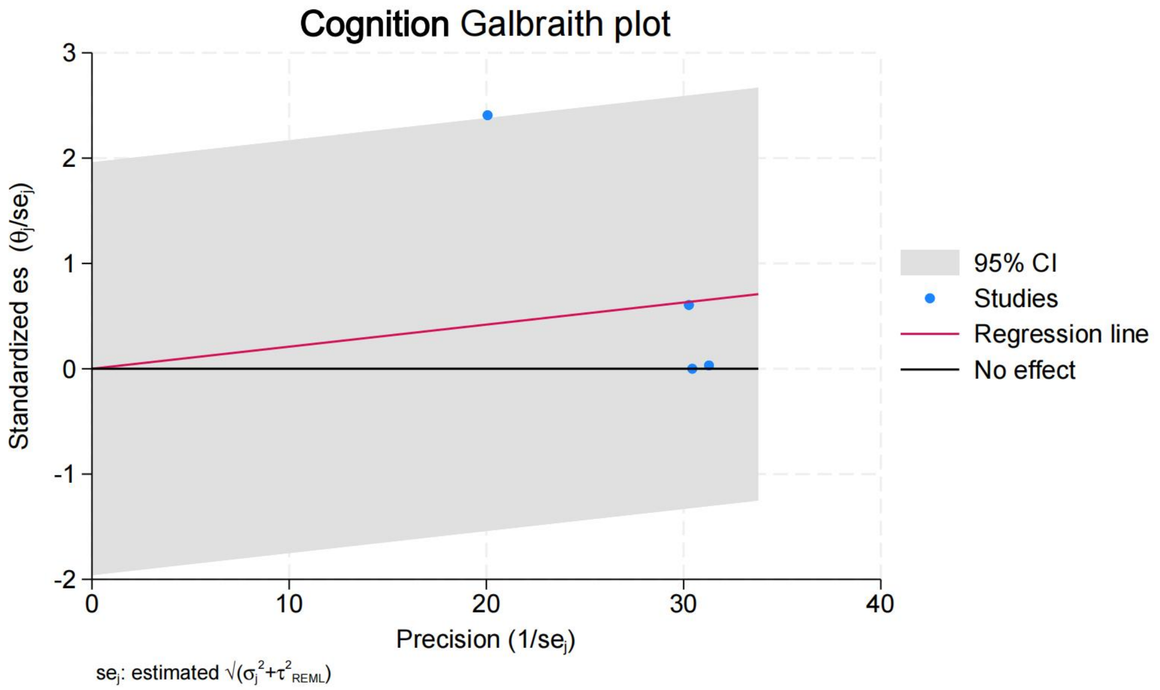

Supplementary Figure 2. The Galbraith plot of meta-analysis. **(A)** Galbraith plot for anxiety. **(B)** Galbraith plot for depression. **(C)** Galbraith plot for stroke. **(D)** Galbraith plot for dementia. **(E)** Galbraith plot for cognition.

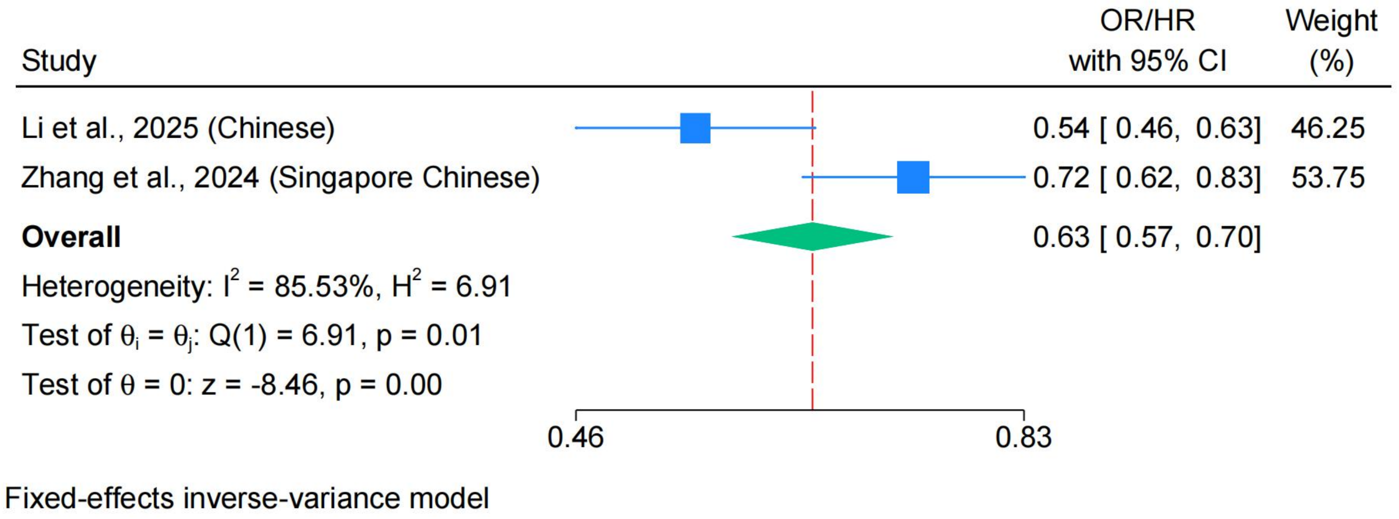

Supplementary Figure 3. The forest plots of cognitive impairment. A direct combination of the two effect estimates was performed despite differences in measure type (OR vs HR). This yielded a pooled estimate of 0.63 (95% CI, 0.57–0.70) and should be interpreted with caution given the methodological incompatibility.

A

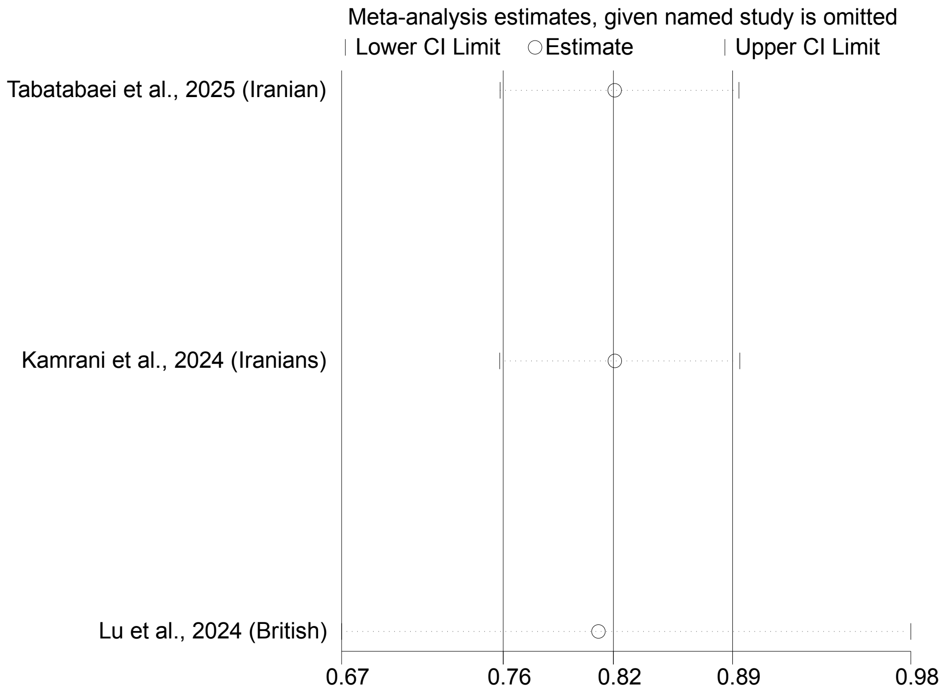

Meta-analysis estimates, given named study is omitted
Lower CI Limit
Estimate
Upper CI Limit
Tabatabaei et al., 2025 (Iranian)
Kamrani et al., 2024 (Iranians)
Lu et al., 2024 (British)
0.67
0.76
0.82
0.89
0.98

B

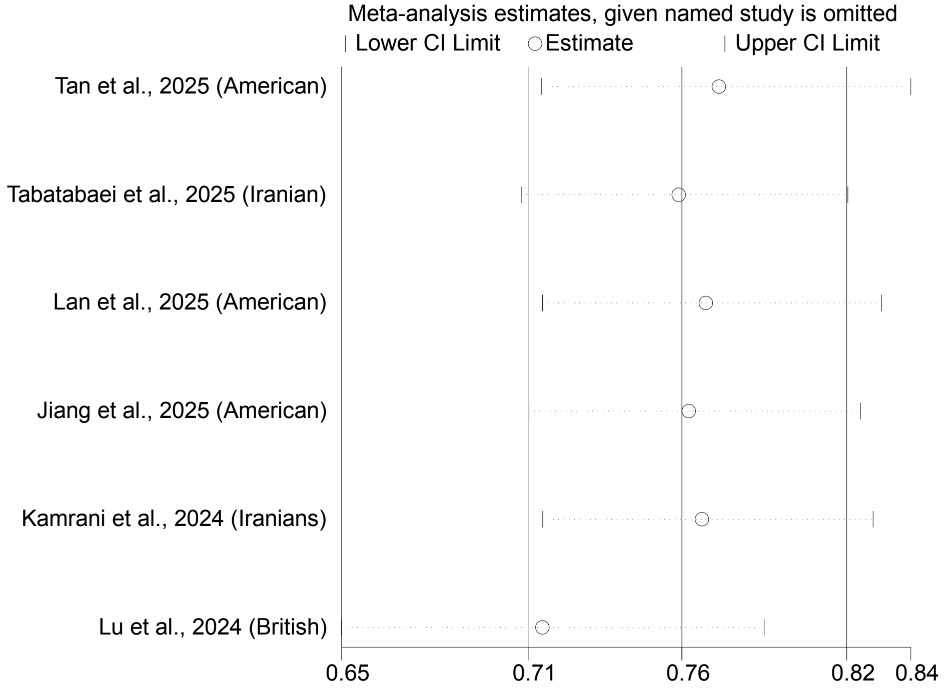

Meta-analysis estimates, given named study is omitted
Lower CI Limit
Estimate
Upper CI Limit
Tan et al., 2025 (American)
Tabatabaei et al., 2025 (Iranian)
Lan et al., 2025 (American)
Jiang et al., 2025 (American)
Kamrani et al., 2024 (Iranians)
Lu et al., 2024 (British)
0.65
0.71
0.76
0.82
0.84

C

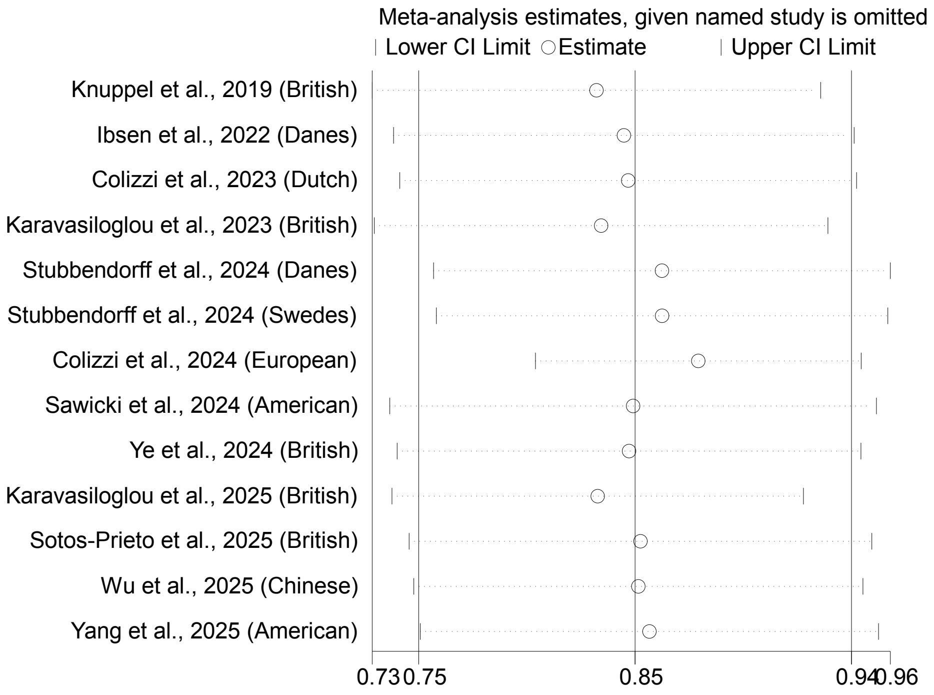
Meta-analysis estimates, given named study is omitted
Lower CI Limit
Estimate
Upper CI Limit
Knuppel et al., 2019 (British)
Ibsen et al., 2022 (Danes)
Colizzi et al., 2023 (Dutch)
Karavasiloglou et al., 2023 (British)
Stubbendorff et al., 2024 (Danes)
Stubbendorff et al., 2024 (Swedes)
Colizzi et al., 2024 (European)
Sawicki et al., 2024 (American)
Ye et al., 2024 (British)
Karavasiloglou et al., 2025 (British)
Sotos-Prieto et al., 2025 (British)
Wu et al., 2025 (Chinese)
Yang et al., 2025 (American)
0.73
0.75
0.85
0.94
0.96

D

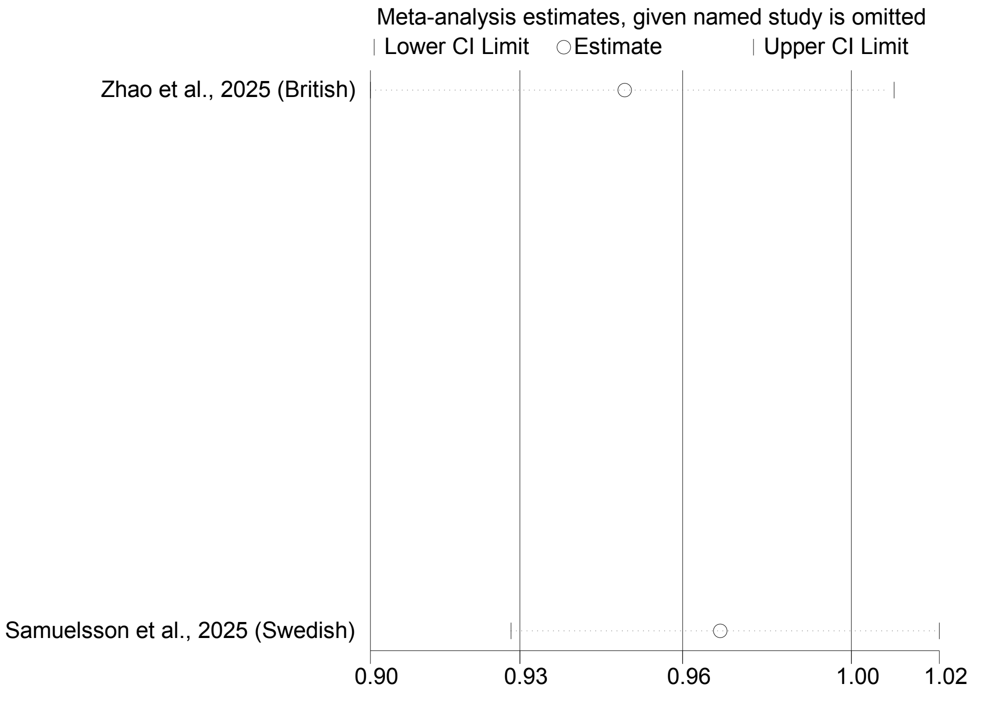
Meta-analysis estimates, given named study is omitted
Lower CI Limit
Estimate
Upper CI Limit
Zhao et al., 2025 (British)
Samuelsson et al., 2025 (Swedish)
0.90
0.93
0.96
1.00
1.02

E

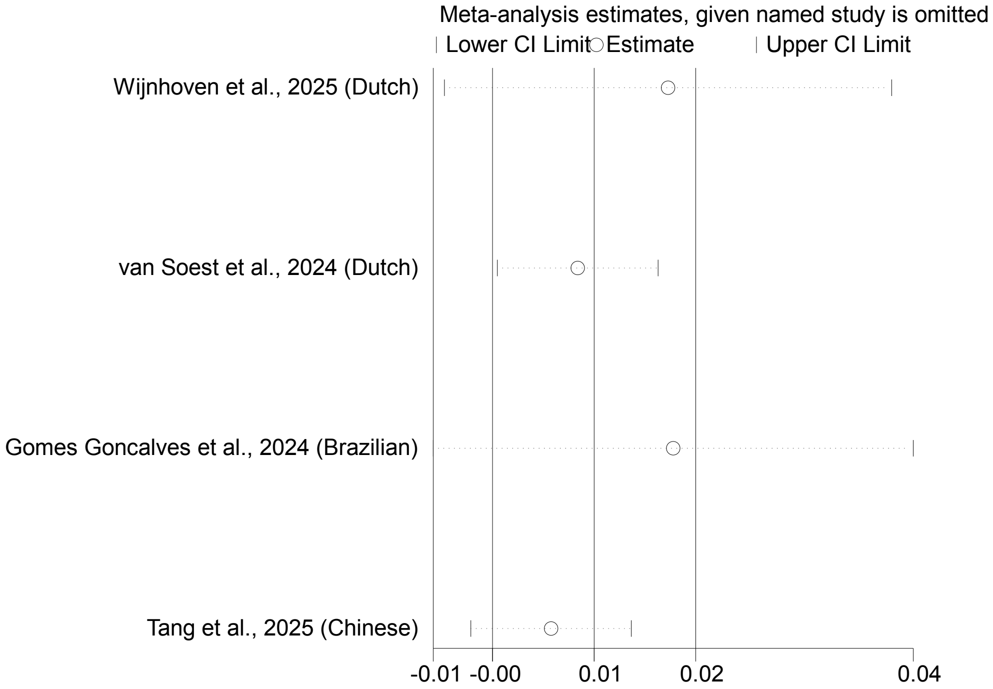

Supplementary Figure 4. Results of sensitivity analysis (influence analyses). (A) Sensitivity analysis for anxiety. **(B)**Sensitivity analysis for depression. **(C)** Sensitivity analysis for stroke. **(D)** Sensitivity analysis for dementia. **(E)** Sensitivity analysis for cognition.

A. Depression

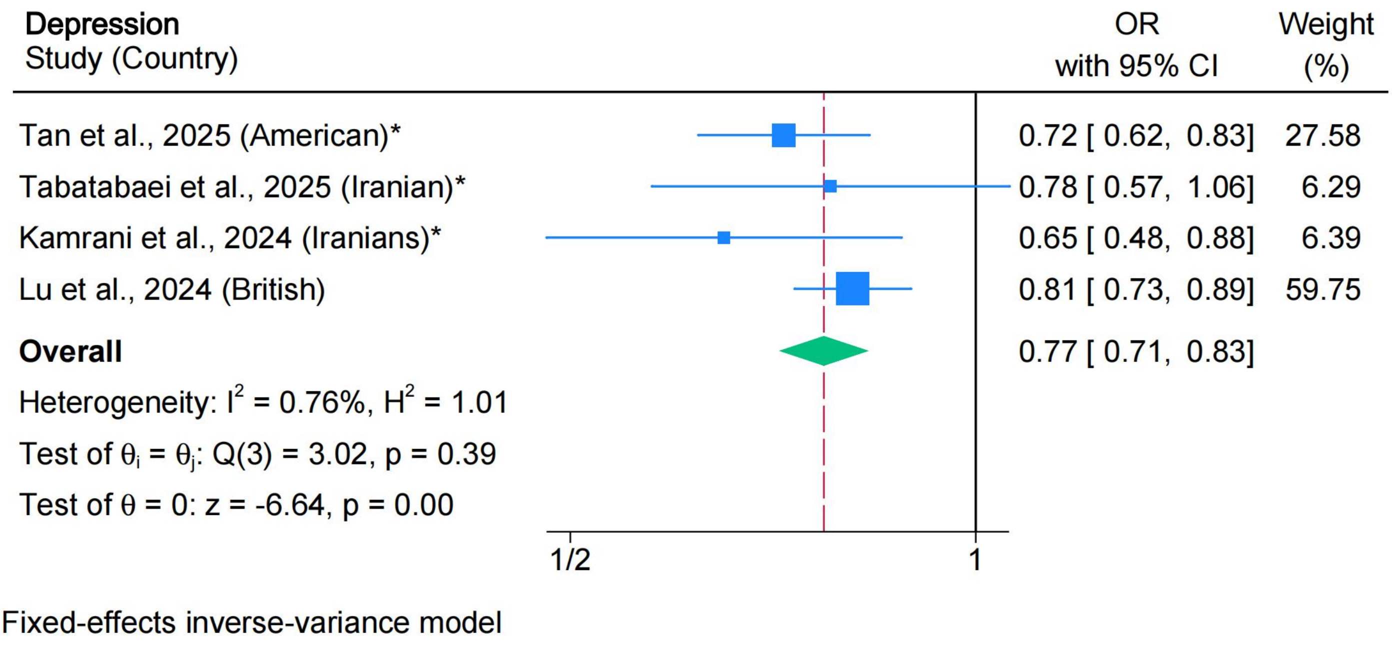

B. Depression

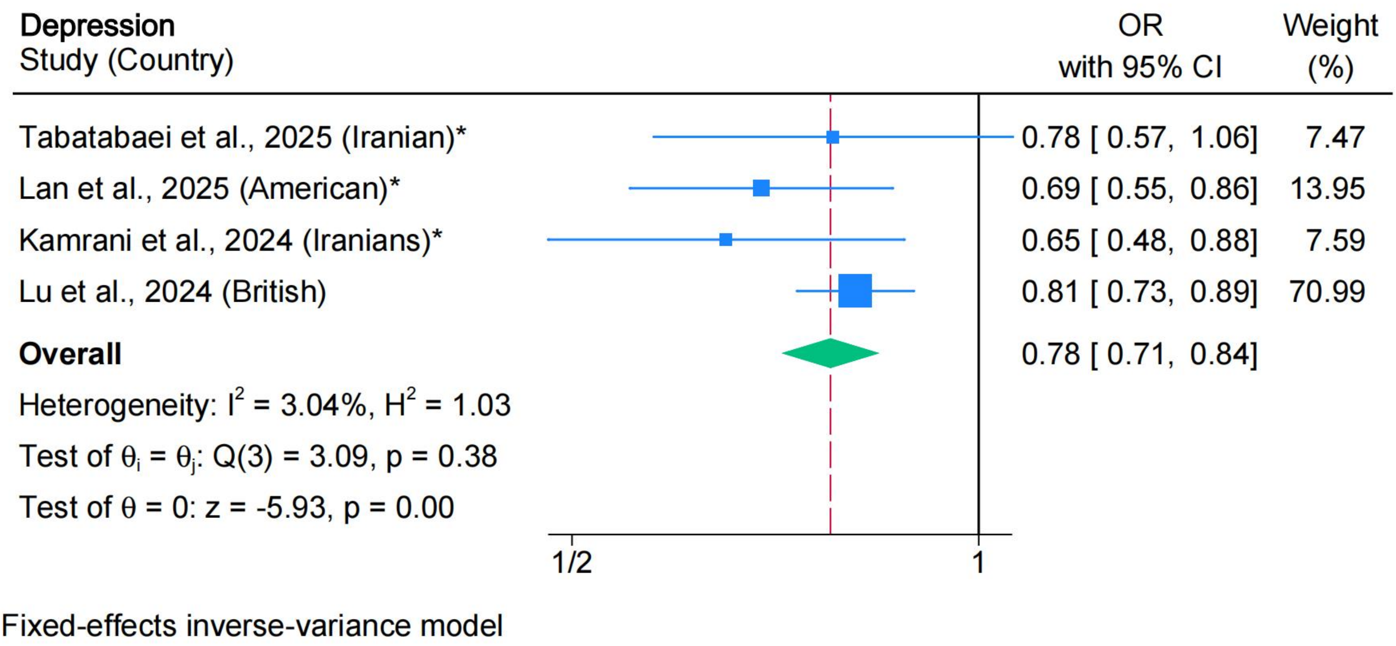

C. Depression

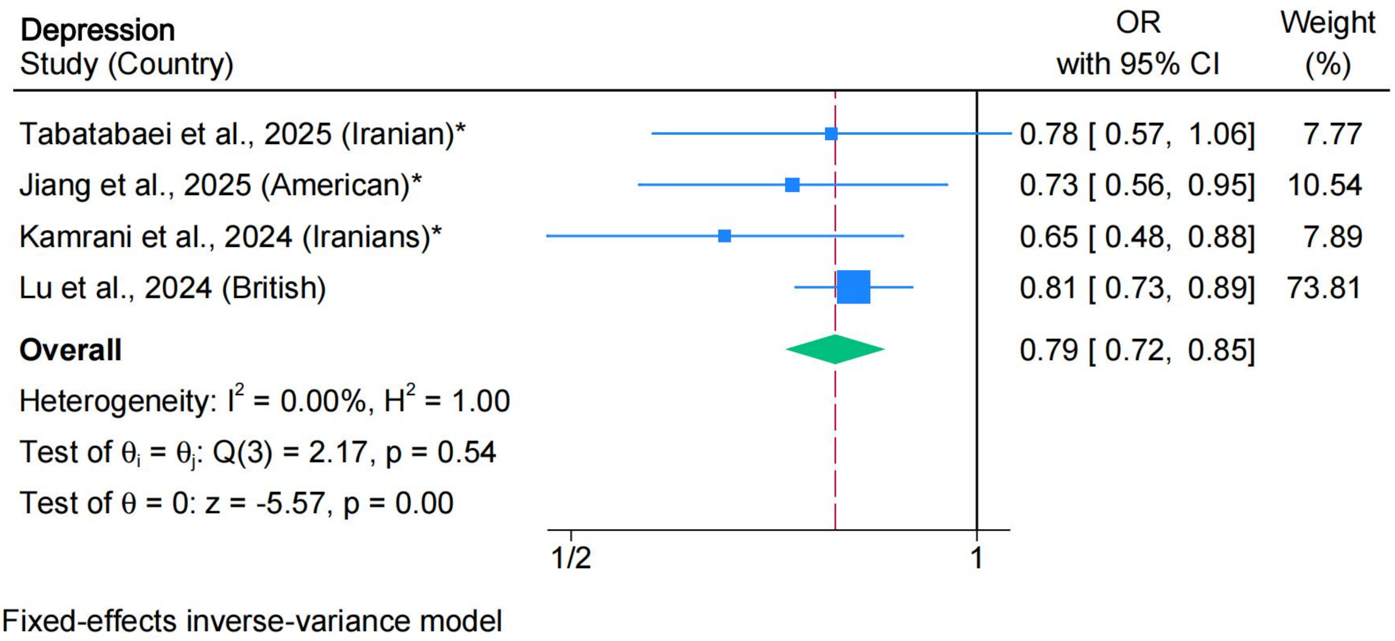

D. Stroke

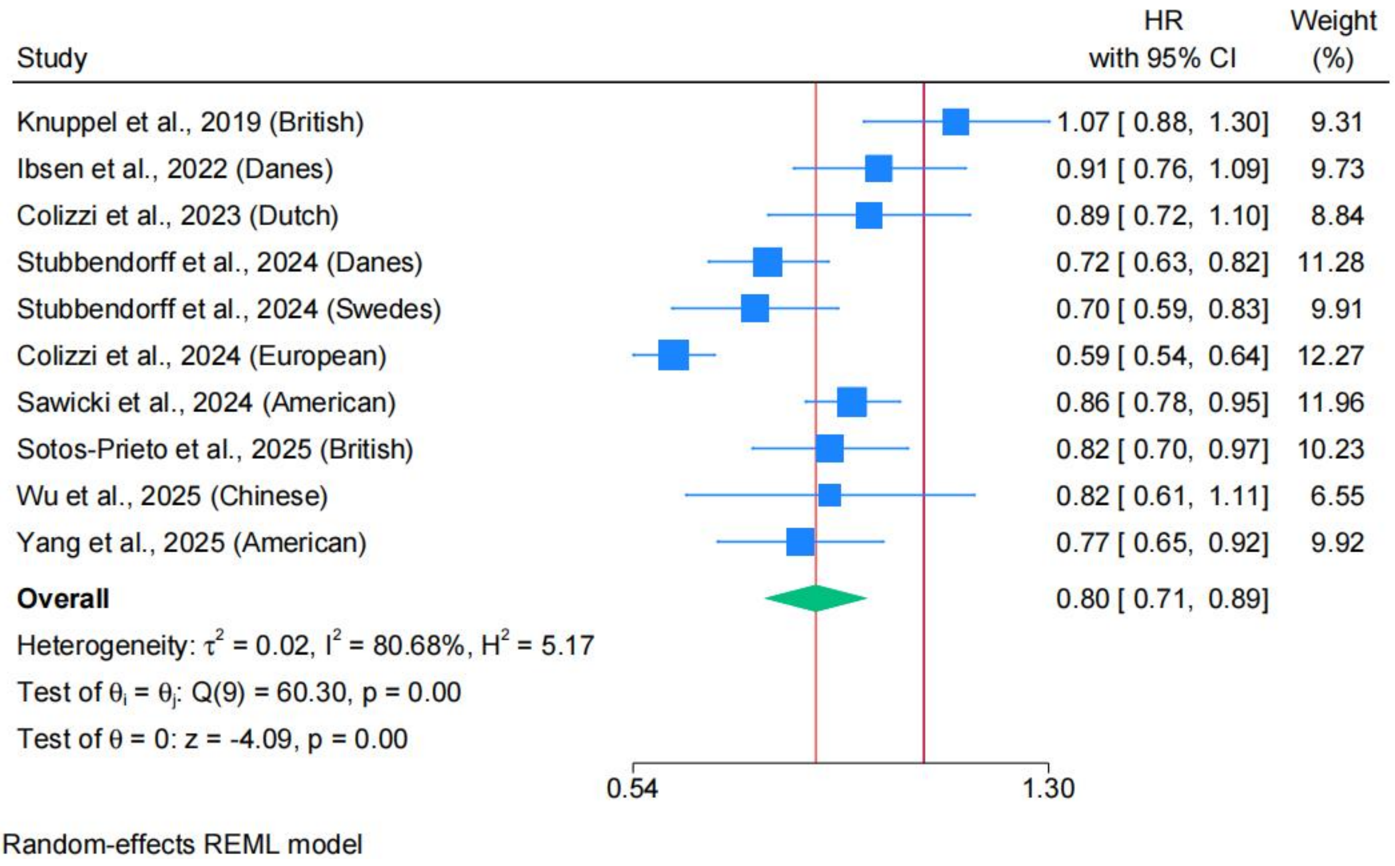

E. Stroke

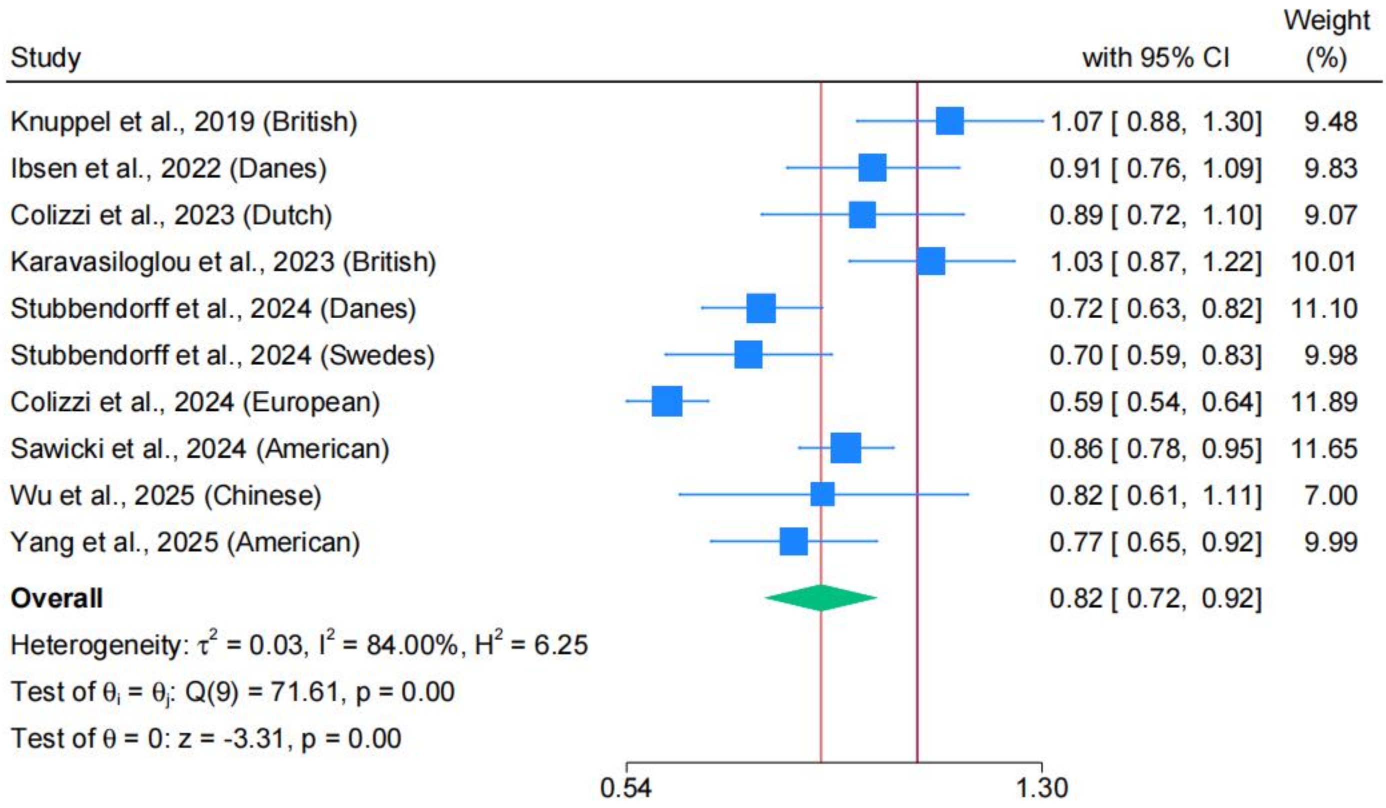

Random-effects REML model

F. Stroke

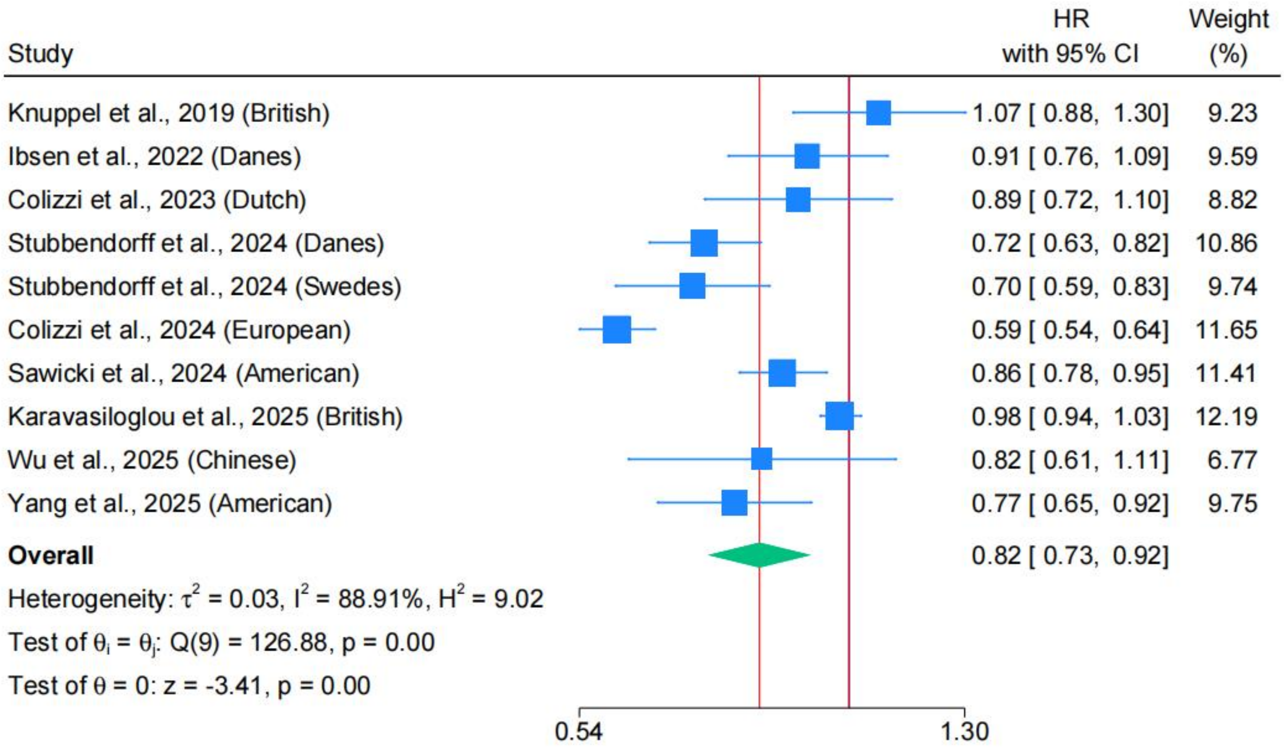

Random-effects REML model

G. Stroke

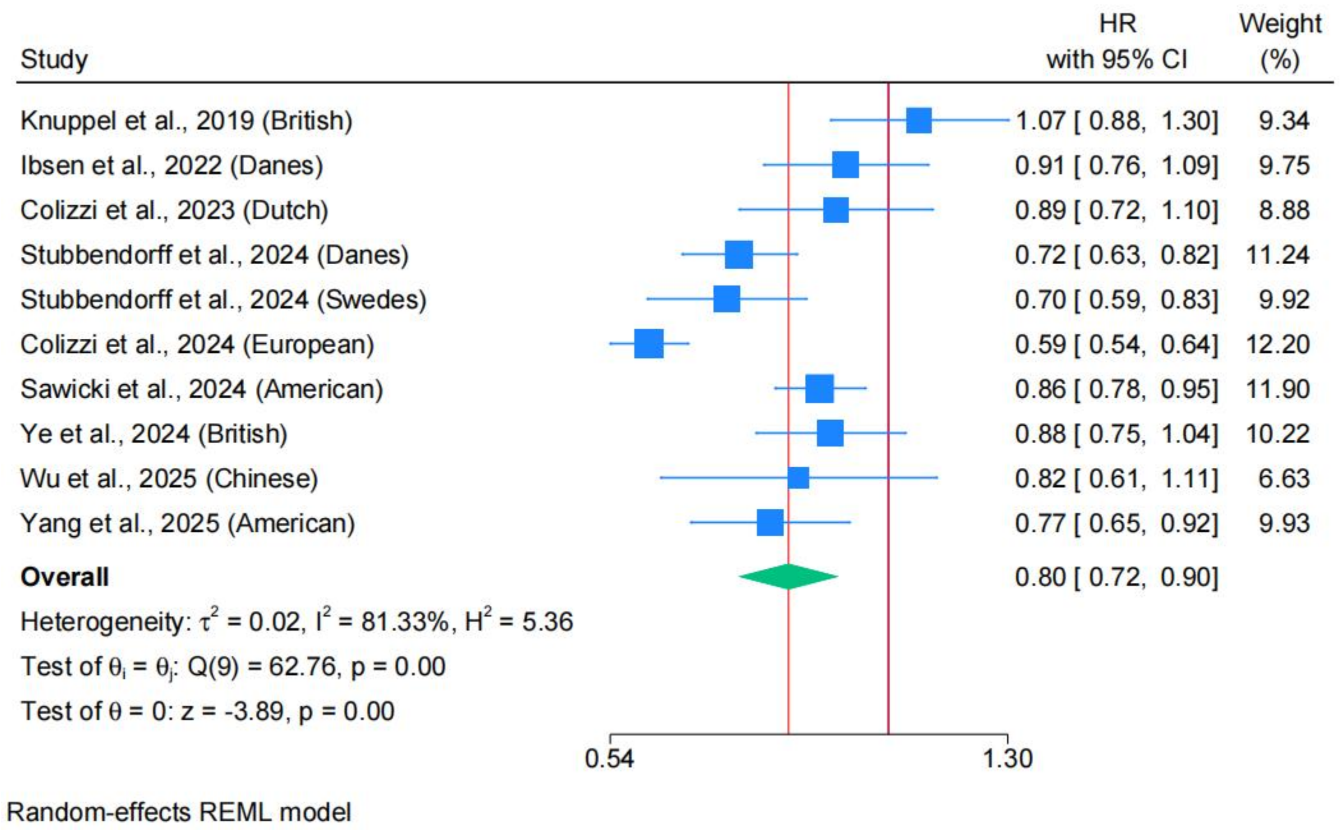

Supplementary Figure 5. Sensitivity analysis assessing the impact of overlapping publications from the same cohort on the pooled estimates. (A) Meta-analysis of depression including only Tan et al., 2025(Tan et al., 2025) from overlapping cohorts. **(B)**Meta-analysis of depression including only Lan et al., 2025(Lan, Chen, Lin, Tang, & Zhang, 2025) from overlapping cohorts. **(C)** Meta-analysis of depression including only Jiang et al., 2025(Jiang, Choi, & Gong, 2025) from overlapping cohorts. **(D)** Meta-analysis of stroke including only Sotos-Prieto et al., 2025(Sotos-Prieto et al., 2025) from overlapping cohorts. **(E)** Meta-analysis of stroke including only Karavasiloglou et al., 2023(Nena Karavasiloglou et al., 2023) from overlapping cohorts. **(F)** Meta-analysis of stroke including only Karavasiloglou et al., 2025(N. Karavasiloglou et al., 2025) from overlapping cohorts. **(G)** Meta-analysis of stroke including only Ye et al., 2024(Ye et al., 2024) from overlapping cohorts.

Reference

Jiang, C., Choi, S., & Gong, H. (2025). From planetary health diet (PHD) to mental health: Higher PHD index protects against depression among the U.S. population. J Psychiatr Res, 183, 31–38. doi:10.1016/j.jpsychires.2025.02.006

Karavasiloglou, N., Suter, F., Thompson, A. S., Pestoni, G., Cassidy, A., Kühn, T., & Rohrmann, S. (2025). Association between habitual adherence to the planetary-health diet and mortality and major chronic disease risk among UK Biobank participants. The American journal of clinical nutrition, 122(3), 755-761. doi:10.1016/j.ajcnut.2025.07.003

Karavasiloglou, N., Thompson, A. S., Pestoni, G., Knuppel, A., Papier, K., Cassidy, A., . . . Rohrmann, S. (2023). Adherence to the EAT-Lancet reference diet is associated with a reduced risk of incident cancer and all-cause mortality in UK adults. One Earth, 6(12). doi:10.1016/j.oneear.2023.11.002

Lan, Y., Chen, L., Lin, Z., Tang, H., & Zhang, X. (2025). Association of planetary health diet index with depression and mortality in the United States. BMC Psychiatry, 25(1), 556. doi:10.1186/s12888-025-06987-x

Pequeno, N. P. F., Cabral, N. L. A., Marchioni, D. M., Lima, S., & Lyra, C. O. (2020). Quality of life assessment instruments for adults: a systematic review of population-based studies. Health Qual Life Outcomes, 18(1), 208. doi:10.1186/s12955-020-01347-7

Sotos-Prieto, M., Ortolá, R., Maroto-Rodriguez, J., Carballo-Casla, A., Kales, S. N., & Rodríguez-Artalejo, F. (2025). Association between planetary health diet and cardiovascular disease: a prospective study from the UK Biobank. Eur J Prev Cardiol, 32(5), 394–401. doi:10.1093/eurjpc/zwae282

Tan, J.-x., Li, Q.-z., Mo, Y.-x., Zhou, H.-p., Miao, L., Ruan, G.-t., . . . Gong, Y.-z. (2025). Evaluating and modifying the PHDI for depression prevention: insights from NHANES 2005-2018. Frontiers in Nutrition, 12. doi:10.3389/fnut.2025.1601129

Wells, G. A., Shea, B., O'Connell, D., Peterson, J., Welch, V., Losos, M., & Tugwell, P. (2000). The Newcastle-Ottawa Scale (NOS) for assessing the quality of nonrandomised studies in meta-analyses.

Willett, W., Rockström, J., Loken, B., Springmann, M., Lang, T., Vermeulen, S., . . . Murray, C. J. L. (2019). Food in the Anthropocene: the EAT-Lancet Commission on healthy diets from sustainable food systems. Lancet, 393(10170), 447-492. doi:10.1016/s0140-6736(18)31788-4

Ye, Y. X., Chen, J. X., Li, Y., Lai, Y. W., Lu, Q., Xia, P. F., . . . Pan, A. (2024). Adherence to a planetary health diet, genetic susceptibility, and incident cardiovascular disease: a prospective cohort study from the UK Biobank. Am J Clin Nutr, 120(3), 648–655. doi:10.1016/j.ajcnut.2024.06.014
